# Randomised controlled phase I trial of a SARS‐CoV‐2 monoclonal antibody

**DOI:** 10.1002/cti2.70126

**Published:** 2026-11-04

**Authors:** Wen Shi Lee, Kevin J Selva, Timothy E Schlub, Jennifer Audsley, Janavi S Rambhatla, Robyn Esterbauer, Jennifer A Juno, Andrew Kelly, Ellie Reilly, Thakshila Amarasena, Sarah Lee, Hyon‐Xhi Tan, Chee Wah Tan, Lin‐Fa Wang, Christina C Chang, Michael Gerometta, Judith A Scoble, Patrick F James, Arnold Reynaldi, Miles P Davenport, Kym Hoger, Karen Hughes, Martina L Jones, Ben Hughes, Adam K Wheatley, Stephen J Kent

**Affiliations:** ^1^ Department of Microbiology and Immunology University of Melbourne, The Peter Doherty Institute for Infection and Immunity Melbourne VIC Australia; ^2^ Faculty of Medicine and Health, Sydney School of Public Health The University of Sydney Sydney NSW Australia; ^3^ Department of Infectious Diseases University of Melbourne, The Peter Doherty Institute for Infection and Immunity Melbourne VIC Australia; ^4^ Programme in Emerging Infectious Diseases Duke‐NUS Medical School Singapore Singapore; ^5^ Infectious Diseases Translational Research Program, Department of Microbiology and Immunology Yong Loo Lin School of Medicine, National University of Singapore Singapore Singapore; ^6^ National Centre for Infectious Diseases Singapore Singapore; ^7^ Nucleus Network Melbourne VIC Australia; ^8^ Department of Infectious Diseases The Alfred Hospital and School of Translational Medicine, Monash University Melbourne VIC Australia; ^9^ BioSensis Consulting Brisbane QLD Australia; ^10^ Biotherapeutics Program CSIRO Biosecurity Clayton VIC Australia; ^11^ Kirby Institute University of New South Wales Kensington NSW Australia; ^12^ National Biologics Facility, Australian Institute for Bioengineering and Nanotechnology The University of Queensland Brisbane QLD Australia; ^13^ Melbourne Sexual Health Centre and Department of Infectious Diseases Alfred Hospital and Central Clinical School, Monash University Melbourne VIC Australia

**Keywords:** monoclonal antibody, neutralisation, pharmacokinetics, phase I, salivary antibody, SARS‐CoV‐2

## Abstract

**Objectives:**

SARS‐CoV‐2 infection remains a threat to vulnerable groups. Neutralising monoclonal antibodies (mAbs) have shown efficacy in the treatment and prevention of COVID‐19, but most mAbs have lost activity against recent Omicron strains.

**Methods:**

We previously isolated a receptor binding domain‐specific mAb from a COVID‐19 convalescent donor, PDI204, which retained activity against several early Omicron strains. This randomised, double‐blind, placebo‐controlled phase I clinical trial enrolled healthy adults (*n* = 32) to receive PDI204 intravenously (IV) at three different doses (200, 600, 1200 mg) or intramuscularly (IM) at 300 mg.

**Results:**

PDI204 was safe and tolerable with only Grade 1 and 2 adverse events. High serum levels of PDI204 were detected in PDI204‐dosed participants with a median elimination half‐life of 17.7 days (IQR 16.3–22.1). Boosted neutralising activity and Fc receptor binding were observed in serum against ancestral and Omicron BA.5 SARS‐CoV‐2 strains, but not against the later Omicron strain XBB.1.5. IM‐dosed subjects had peak PDI204 levels in serum 2–6 days later than IV‐dosed subjects. Entry of PDI204 into saliva from blood was low and variable, and primarily detected in participants receiving 600 mg or 1200 mg doses. Three of 32 people acquired COVID‐19 within 90 days of dosing, two receiving PDI204 and one in a placebo recipient.

**Conclusion:**

Overall, the mAb PDI204 was safe but lost activity against later Omicron strains and did not prevent COVID‐19 during this trial.

## Introduction

New treatments to prevent and treat COVID‐19 are needed, particularly for the elderly and immunosuppressed. The initial efficacy of small molecule antivirals appears to have reduced over time.^1^ Neutralising monoclonal antibodies against spike of SARS‐CoV‐2 were highly effective early in the COVID‐19 pandemic,^2^ either given intramuscularly as long‐acting prophylactics to prevent infection,^3^, ^4^ or intravenously to treat infection.^5^, ^6^, ^7^ However, SARS‐CoV‐2 evolved to escape antibody immunity, and most mAbs have subsequently been withdrawn from clinical use because of the expansion and subsequent dominance of neutralisation‐escaped variants.^8^, ^9^ In particular, Omicron strains emerged in late 2021 with many neutralisation‐escape mutations in the receptor binding domain (RBD) of spike.^10^ Current circulating lineages, including XFG, NB.1.8.1 and BA.3.2, have continued to acquire spike mutations associated with neutralisation escape,^11^ illustrating the continued challenge of developing broadly neutralising mAb therapeutics that are resistant to viral escape.

We generated a panel of human SARS‐CoV‐2 IgG1 mAbs from memory B cells of SARS‐CoV‐2 convalescent individuals in 2020.^12^ Some mAbs showed high potency and retained neutralisation activity against early SARS‐CoV‐2 variants of concern. One of the most potent mAbs, PDI204, binds to the receptor binding motif of RBD and blocks spike from binding to the ACE2 receptor, thereby neutralising SARS‐CoV‐2 virions. PDI204 could protect mice from Omicron BA.1 challenge when delivered either as protein or as mRNA encapsulated within lipid nanoparticles.^13^ PDI204 belongs to same public class of RBD‐specific antibodies as Tixagevimab (1 of 2 mAbs in the Evusheld cocktail developed by AstraZeneca),^14^ encoded by the heavy‐chain variable germline gene IgHV1‐58 and light‐chain variable germline gene IGKV3‐20. Promising pre‐clinical results against early Omicron variants supported the clinical development of PDI204 as a therapeutic agent for COVID‐19 to treat infection. After toxicology testing and GMP manufacturing of PDI204 as an IgG1 mAb with unmodified Fc, we advanced PDI204 through to a randomised, double‐blinded, human phase I trial in 32 healthy volunteers.

## Results

### PDI204 was safe and well tolerated

Thirty‐two individuals were recruited between June and September 2025 in Melbourne, Australia, and followed for 89 days after infusion (CONSORT diagram shown in Figure 1). Inclusion criteria included male or female, aged between 18 and 65 years and healthy as defined by the absence of clinically significant illness and surgery within 4 weeks prior to study drug administration. Exclusion criteria included clinically significant abnormal laboratory test results at screening and day of clinic confinement (1 day prior to study drug administration) and positive tests for hepatitis B, hepatitis C or HIV (full trial protocol in Supplementary file 1). After appropriate screening, four groups of healthy adult volunteers (6 per group) received a single dose of PDI204, either as an intravenous infusion at 200 mg, 600 mg or 1200 mg doses, or intramuscularly at 300 mg. Two participants received saline placebo concurrently with each of the four groups, for a total of eight participants receiving placebo. Median age of the participants was 33 years and 46% of the cohort was male, with additional demographic information shown in Supplementary table 1. The trial schema is outlined in Supplementary figure 1.

**Figure 1:**
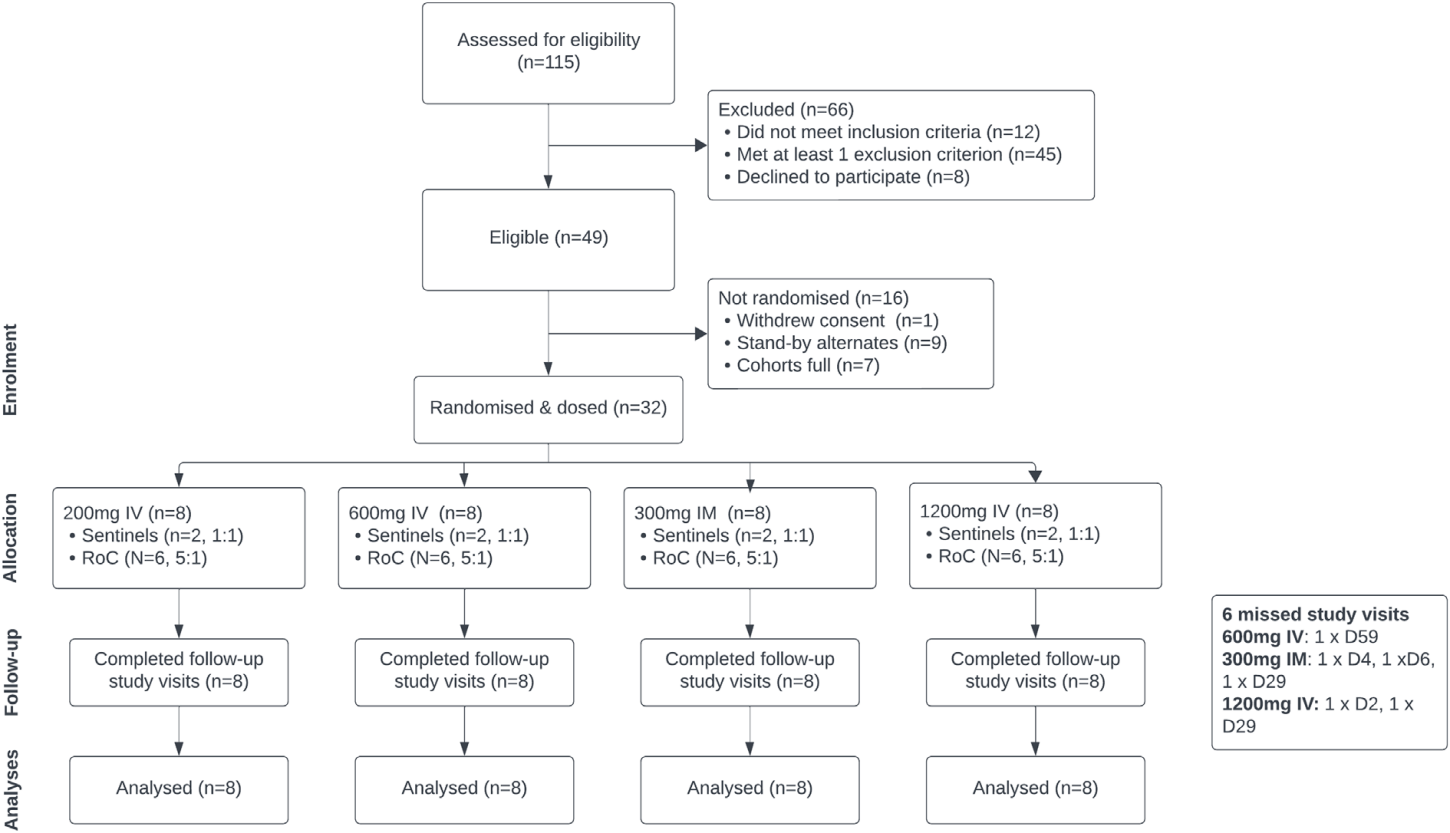
CONSORT flow diagram describing recruitment for the PDI204 trial.

There were 23 Grade 1 or 2 treatment‐emergent adverse events (TEAEs) experienced by 17 participants (53.1% of the cohort), of which only three were deemed probably treatment‐related (Table 1). There were no serious adverse events or TEAEs greater than Grade 2. Grade 1 administration site conditions were the most common adverse events. Twelve participants experienced one TEAE, three experienced two TEAEs, and two participants experienced three TEAEs. There were no adverse events related to vital signs and no clinically significant 12‐lead standard ECG findings (Supplementary table 2). Study laboratory adverse events were uncommon and resolved quickly among three panels (biochemistry, haematology and urinalysis) of 57 analytes measured at seven post‐treatment visits (Supplementary table 2). All participants tested negative for anti‐PDI204 anti‐drug antibodies (ADA) at three timepoints (pre‐dose, Day 29 and Day 89).

**Table 1:** Treatment‐emergent adverse events (TEAEs)

TEAE classification	Total treated cohort (*n* = 24)	IM 300 mg (*n* = 6)	IV 200 mg (*n* = 6)	IV 600 mg (*n* = 6)	IV 1200 mg (*n* = 6)	Placebo^a^ (*n* = 8)	TEAE grade	Related to IP
Any event	12 (18) [50.0]	3 (9.38) [6]	3 (9.38) [5]	4 (12.5) [5]	2 (6.25) [2]	4 (12.5) [5]		
Blood & lymphatic system disorders	1 (4.2) [1]							
Neutropenia	1	1					Gr 2	Remote/unlikely
Gastrointestinal								
Abdominal discomfort						1	Gr1	Placebo
General disorders & administration site conditions	5 (20.8) [5]							
Injection site reaction erythema	2		1	1			Gr1	1 × probably; 1 × unrelated
Infusion site reaction bruising	1			1			Gr1	Probably
Injection site reaction bruising	1	1					Gr1	Probably
Bruising secondary to IV cannula	1				1		Gr1	Remote/unlikely
Infections & infestations	2 (8.3) [3]							
Gastroenteritis	1				1		Gr2	Remote/unlikely
COVID‐19	1	1					Gr1	Unrelated
Herpes labialis	1	1					Gr1	Unrelated
Investigations	1 (4.2) [2]							
ALT increase	1		1				Gr1	Possibly
AST increase	1		1				Gr1	Possibly
Lipase increase						1	Gr2	Placebo
Musculoskeletal & connective tissue disorders	1 (4.2) [1]							
Subacromial bursitis	1			1			Gr2	Unrelated
Back pain						1	Gr1	Placebo
Nervous system disorders	1 (4.2) [1]							
Headache	1	1				1	Gr1	1 × possibly; 1 × placebo
Light headedness						1	Gr1	Placebo
Respiratory, thoracic & mediastinal disorders	1 (4.2) [1]							
Allergic rhinitis	1	1					Gr2	Unrelated
Reproductive system & breast disorders	2 (8.3) [2]							
Dysmenorrhea	1		1				Gr1	Unrelated
Late period	1			1			Gr1	Remote/unlikely
Skin & subcutaneous tissue disorders	2 (8.3) [2]							
Contact dermatitis			1	1			Gr1	2 × unrelated

Results are presented as the number of participants with the event, the proportion of participants with the event (%) and the number of events [e].

Gr, Grade; IM, intramuscular; IP, investigational product; IV, intravenous; TEAE, treatment‐emergent adverse event.

^a^ Placebo group not included in ‘total treated cohort’ numbers.

### PDI204 pharmacokinetics in serum and saliva

We evaluated serial serum and saliva samples for pharmacokinetics of PDI204. PDI204 concentration generally peaked in serum within 4 h of IV dosing (median *T*_max_ of 0.5 h for all doses) while it was slightly delayed for IM dosing, peaking between 2 and 6 days (median *T*_max_ of 120 h) (Figure 2a; Supplementary figure 2a; Table 2). Higher doses in the IV groups led to expected dose‐proportional increases in peak serum levels of PDI204. Interestingly, the IM 300 mg group had lower peak serum levels of PDI204 compared to the IV 200 mg group (geometric mean *C*_max_ of 22.3 μg/mL and 79.1 μg/mL respectively), though PDI204 serum levels equalised in these two groups from Day 14 onwards (Figure 2a). The median serum half‐life (T_1/2_) of PDI204 was 17.6 days (IQR 16.3–22.1) across all dosed participants, and was similar across groups (Table 2), consistent with PDI204 being a fully human IgG1 mAb.^15^

**Figure 2:**
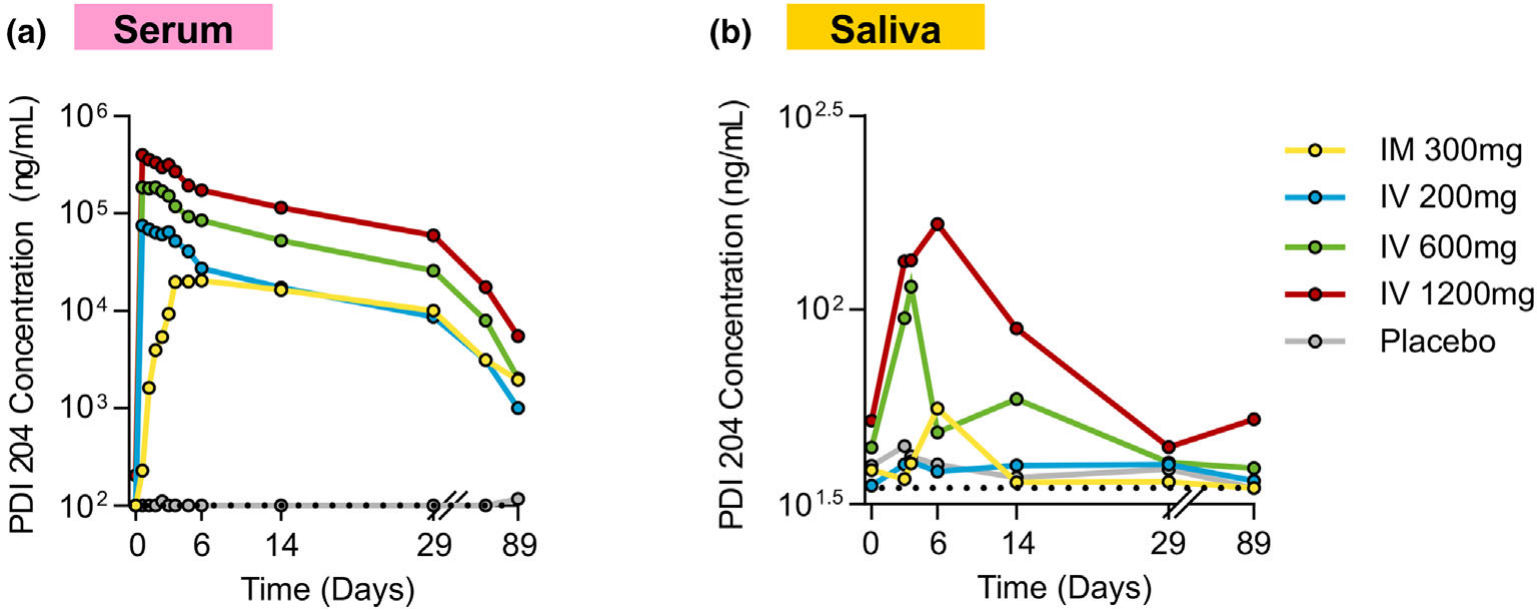
Pharmacokinetics of PDI204 in serum and saliva. Mean concentrations of PDI204 measured in **(a)** serum and **(b)** saliva at respective timepoints for the different treatment cohorts (*n* = 6 for each IV and IM group, *n* = 8 for placebo group). PDI204 concentration was measured via capture ELISA for serum and a bead‐based multiplex assay for saliva. Dotted lines indicate the limit of detection. Assays were performed in duplicate.

**Table 2:** Pharmacokinetics of intravenous (IV) or intramuscular (IM) administered PDI204

Cohort	Geo. mean *C*_max_ (μg/mL)	Mean *T*_max_ (h)	Median *T*_max_ (h)	Mean k_el_ (day^−1^)	Mean T_1/2_ (day)	Median T_1/2_ (day)	Geo. mean AUC_0–t_ (h*μg/mL)	Geo. mean AUC_0–inf_ (h*μg/mL)	Geo. mean residual AUC (%)	Mean CL (mL/h)	Mean V_z_ (L)
IM 300 mg	22.3	112	120	0.0380	19.9	21.3	17 638	18 671	4.9	22.20	12.03
IV 200 mg	79.1	4.4	0.5	0.0362	22.6	21.9	22 481	23 897	3.0	8.60	6.50
IV 600 mg	193.9	3.7	0.5	0.0422	16.9	16.4	60 771	62 211	1.9	9.84	5.70
IV 1200 mg	403.4	1.7	0.5	0.0399	17.9	16.8	133 022	136 983	2.4	9.10	5.64
All				0.0391	19.3	17.6					
Placebo	0.1	2136	2136	NA	NA	NA	NA	NA	NA	NA	NA

Across the IV dose range, AUC_0–inf_ increased approximately dose proportionally. The slope of the log–log relationship between AUC_0–inf_ and dose was 0.97 (95% CI 0.79–1.14), with little evidence that clearance differed across doses (*P* = 0.80). IM bioavailability was estimated from log‐transformed clearance values to be 55.4% (95% CI 36.2–85.0%), indicating that in the IM group, approximately 55% of the systemic availability from an equivalent IV dose is expected. This was consistent with the similar but slightly lower AUC_0–inf_ following 300 mg IM compared with 200 mg IV, despite the higher IM dose (Supplementary figure S2). Given the approximate dose proportionality, the IV cohorts were pooled after dose normalisation to examine the relationship with body weight. Dose‐normalised AUC_0–inf_ decreased by 7.3% per 10 kg increase in weight (95% CI 1–13%; *P* = 0.029). A similar trend was observed in the IM cohort, although there was little evidence of an association (*P* = 0.88), with interpretation limited by the small sample and single IM dose.

To evaluate how rapidly PDI204 was detectable in upper respiratory mucosal surfaces, the key portal of entry for SARS‐CoV‐2, we measured pharmacokinetics in serial saliva samples. PDI204 was detectable in saliva in many participants, particularly at higher doses (1200 mg and 600 mg IV groups, at mean peak concentrations of 222 ng/mL and 115 ng/mL respectively). Nevertheless, the levels of PDI204 were on average 1800‐fold lower than serum levels and were variable between participants (Figure 2b; Supplementary figure 2b). Although we sampled saliva PK at fewer early timepoints compared to serum, there was generally a delay between the early peak of PDI204 in serum compared to the peak in saliva, consistent with the slow transudation of IgG into saliva.

### PDI204 administration increased SARS‐CoV‐2 neutralising activity in serum and saliva

Neutralising antibodies are a key correlate of protection from infection and progressive disease.^16^, ^17^ We tested serial serum and saliva samples for neutralisation of the ancestral SARS‐CoV‐2 strain, an early Omicron strain (BA.5) and a later Omicron strain XBB.1.5. Neutralising activity was tested using a live virus assay for serum and an RBD‐ACE2 inhibition assay for saliva (Figure 3). Baseline serum neutralising antibody levels (prior to PDI204 administration) varied between participants, consistent with likely diverse infection and vaccination histories, and were generally higher at baseline against ancestral and BA.5 variants compared to that against XBB.1.5 (Figure 3a).

**Figure 3:**
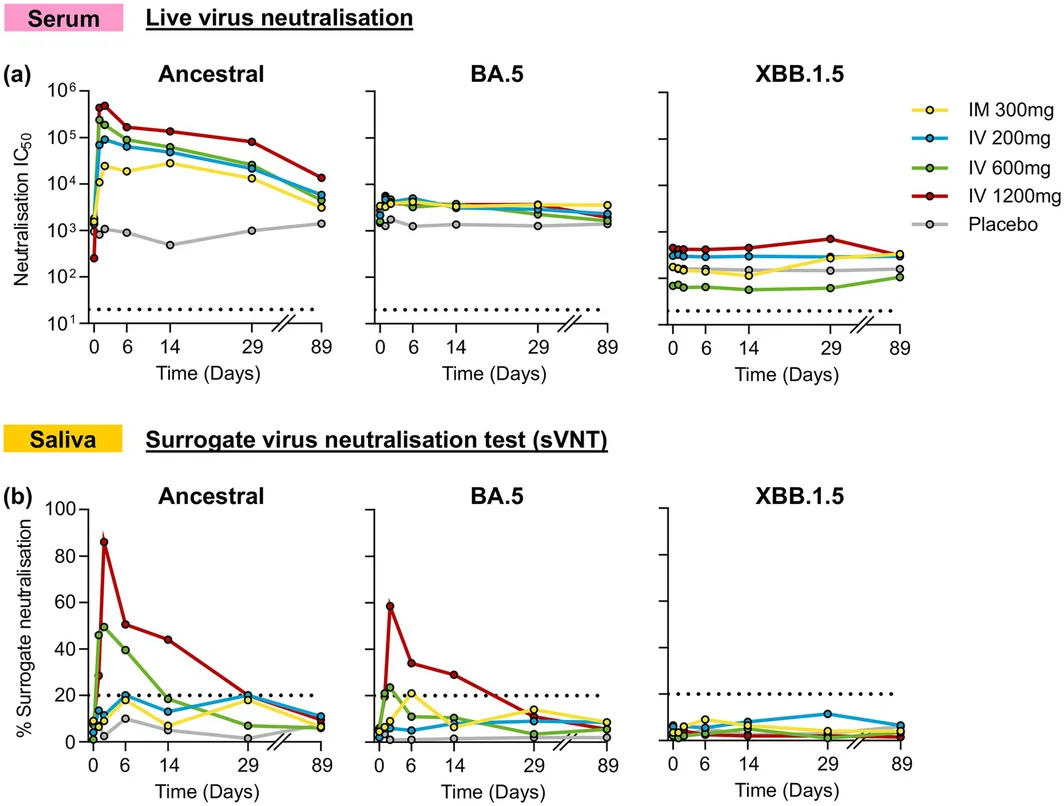
Neutralising activity in serum and saliva. **(a)** Serum and **(b)** saliva neutralising responses against ancestral, BA.5 and XBB.1.5 SARS‐CoV‐2 strains across timepoints for all cohorts using live virus and surrogate neutralisation assays respectively. Dotted lines indicate the cut‐off values of the respective assays. Median responses are displayed for each cohort (*n* = 6 for each IV and IM group, *n* = 8 for placebo group).

After PDI204 administration, there was a clear dose‐proportional rise in serum neutralising activity against the ancestral strain, a moderate rise against the Omicron BA.5 strain, but no increase against the Omicron XBB.1.5 strain (Figure 3a, Supplementary figure 4a, Supplementary table 3), indicating escape from PDI204 neutralisation by more recent Omicron strains. Rises in neutralisation against ancestral SARS‐CoV‐2 were more pronounced in the higher dose groups but were readily detectable at all doses and routes of administration.

After PDI204 administration, surrogate neutralising activity in saliva also increased against ancestral SARS‐CoV‐2 and Omicron BA.5, but not to Omicron XBB.1.5 (Figure 3b, Supplementary figure 4b). Consistent with the PK analyses above, the peak saliva neutralising activity was generally delayed and more variable compared to that observed in serum.

### PDI204 in serum and saliva boosts Fc receptor engagement

There is interest in SARS‐CoV‐2 spike‐specific antibodies that engage Fc receptors and mediate functions such as antibody‐dependent cellular cytotoxicity (ADCC, usually through FcγRIIIa) and antibody‐dependent phagocytosis (ADP, usually through FcγRIIa). We evaluated serial serum and saliva samples for their ability to engage FcγRIII and FcγRII in a multiplex assay format (Figure 4; Supplementary figure 5). We found marked rises in spike‐specific FcγRIIIa‐ and FcγRIIa‐engaging antibodies to ancestral and BA.2 SARS‐CoV‐2 strains, but no increased binding to XBB.1.5 strains.

**Figure 4:**
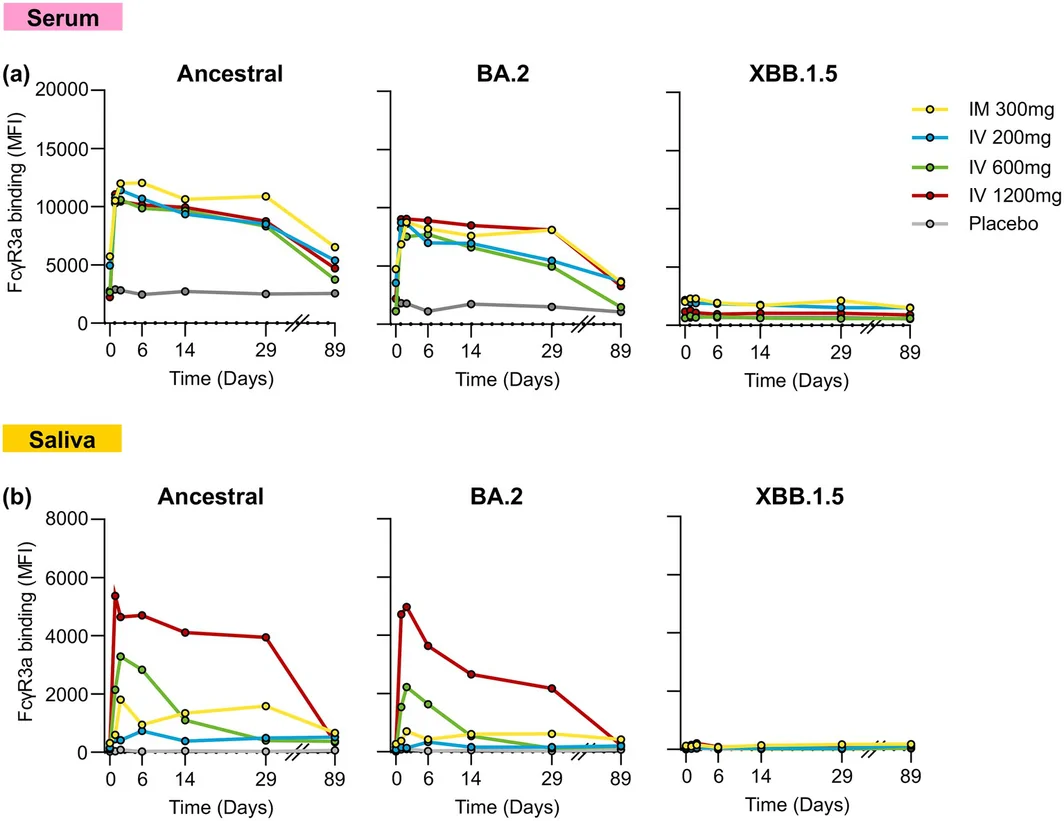
FcγR3a binding responses in serum and saliva. Binding of ancestral, BA.2 and XBB.1.5 spike‐specific antibodies in **(a)** serum and **(b)** saliva to FcγR3a across timepoints for all cohorts, measured via a bead‐based multiplex assay. Dotted lines indicate the background responses of pre‐pandemic samples. Median responses displayed for each cohort (*n* = 6 for each IV and IM group, *n* = 8 for placebo group). Assays were performed in duplicate.

### Breakthrough SARS‐CoV‐2 infections

We followed participants for 89 days after PDI204 or placebo administration. SARS‐CoV‐2 spike‐specific IgG antibodies in serum and saliva waned with time, in line with the longitudinal decay of PDI204 (Supplementary figure 6). One participant reported SARS‐CoV‐2 infection between Days 14 and 29 after 300 mg IM dosing, after performing a rapid antigen test following infection of a close contact. We observed rises in XBB.1.5 neutralising antibodies in this participant and two additional participants between Days 29 and 89 after dosing (from the IV 600 mg and placebo groups) (Figure 5).

**Figure 5:**
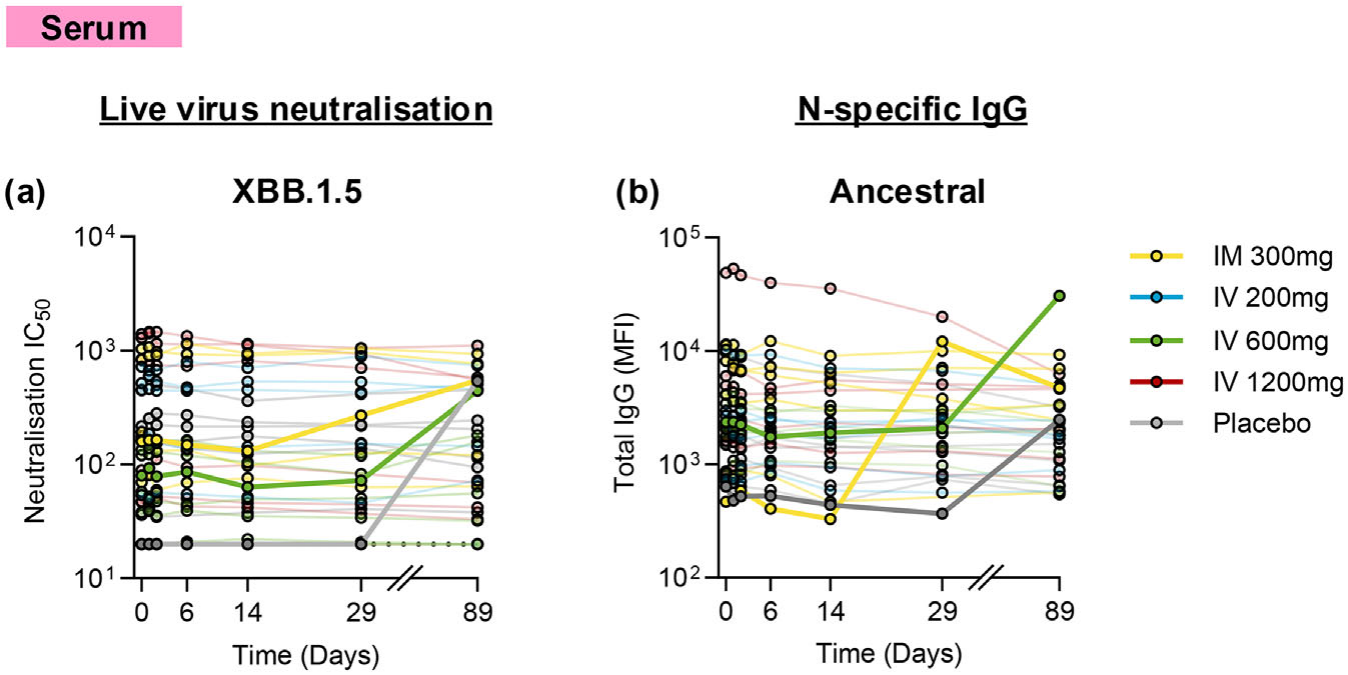
Breakthrough COVID‐19. **(a)** Serum neutralising responses against XBB.1.5 live SARS‐CoV‐2 virus. **(b)** Total IgG binding to ancestral nucleocapsid (N) measured via a bead‐based multiplex assay. Lines show the responses for each individual across timepoints for all cohorts (*n* = 6 for each IV and IM group, *n* = 8 for placebo group). Thicker lines indicate individuals with detectable breakthrough infection. Dotted line indicates the cut‐off value for the live virus neutralisation assay.

We therefore also tested all samples for antibody responses to SARS‐CoV‐2 nucleocapsid (N), a conserved internal protein. The same three participants had greater than fourfold rises in N responses between the same time points, concurrent with the increase in XBB.1.5 neutralising titres. Given that PDI204 does not neutralise XBB.1.5, a rise in both XBB.1.5 neutralising titres and N‐specific IgG responses indicates that these three participants caught SARS‐CoV‐2 infection during the trial.

## Discussion

In this phase I trial, we assessed the safety and tolerability of intravenous and intramuscular administration of a SARS‐CoV‐2 RBD‐specific mAb, PDI204. All 32 participants completed the trial with no serious adverse events. High serum levels of PDI204 were achieved in all participants with a clear dose–response relationship. Saliva levels of PDI204 were more variable between participants and only reliably detected in the higher dose groups (1200 mg and 600 mg IV) at an average of 1800‐fold lower than in serum. Samples from PDI204‐dosed subjects were unable to neutralise a more recent Omicron strain (XBB.1.5), and further clinical development of PDI204 is not proceeding. This highlights the problem of immune escape from serially evolving SARS‐CoV‐2 strains and the need to identify potent neutralising mAbs targeting more conserved epitopes.^18^, ^19^

To our knowledge, this is among the first clinical studies to characterise the pharmacokinetics of a therapeutic mAb in both serum and saliva following IV and IM administration. While previous studies have demonstrated upper‐airway penetration of therapeutic mAbs into the nasal mucosa,^20^, ^21^, ^22^ data describing the kinetics of antibody appearance within saliva are more limited. Our findings provide direct evidence that systemically administered IgG can reach the oral cavity albeit at substantially lower concentrations than in serum. This information could be useful when using mAbs to protect against, or limit the spread of, mucosal pathogens, where protection at the portal of viral entry could contribute to prevention of infection. Our analyses of saliva PDI204 concentrations showed much higher variability compared to serum PDI204 concentrations, likely in part because of the lower concentrations of IgG found in saliva, and physiological and sampling differences across time. Although plasma is a reliable measure of mAb delivery, other mucosal sampling techniques such as nasosorption should be assessed in future trials to gauge mucosal mAb levels. Furthermore, IgA isotypes, particularly dimeric IgA, typically are more potent at neutralising viruses at mucosal surfaces.^23^, ^24^ Future studies should therefore investigate the delivery of IgA mAbs to mucosal surfaces and its protective efficacy against respiratory viruses.

The SARS‐CoV‐2 epidemic continues, although the severity of infections is generally milder since there is a degree of population immunity from prior vaccination and infection.^25^, ^26^ In addition, serial evolution of SARS‐CoV‐2 may have rendered some strains less pathogenic.^27^ None‐the‐less, continued spread of SARS‐CoV‐2 allows opportunities for further immune evasion.^28^, ^29^ In our study, we documented that three of 32 healthy adults acquired SARS‐CoV‐2 infection during the 90 days of follow‐up. Although these are small numbers, this would translate to an annual incidence of infection of 38%. The infections we documented were spread across PDI204 and placebo recipients, suggesting minimal protection mediated by PDI204, consistent with the lack of neutralisation of recent Omicron strains.

In conclusion, PDI204 was safe and well‐tolerated following both IV and IM administration, achieving high systemic concentrations and detectable levels in saliva. Although PDI204 no longer neutralised contemporary Omicron variants and breakthrough infections occurred during the trial, these findings illustrate the challenges posed by ongoing SARS‐CoV‐2 antigenic evolution. Importantly, this study provides clinically relevant pharmacokinetic data on the distribution of therapeutic mAbs from blood to the oral mucosa, which may inform dosing strategies for future mAb therapeutics targeting respiratory and other mucosal pathogens.

## Methods

### PDI204 mAb

PDI204 was isolated from spike and RBD‐binding peripheral blood memory B cells of a subject who recovered from hospitalised COVID‐19 in 2020.^12^ PDI204 was stably expressed in a clonal Chinese Hamster Ovary (CHO) cell line (CHOK1SV GS‐KO®, Lonza) developed by the National Biologics Facility (University of Queensland, Brisbane, Australia). PDI204 drug substance was manufactured at the CSIRO National Vaccine and Therapeutics Laboratory (Clayton, Victoria, Australia) using a multi‐stage purification process including three chromatography steps and two dedicated, orthogonal viral reduction steps. PDI204 was formulated at a concentration of 100 mg/mL and aseptically dispensed into 2 mL vials at Sypharma (Melbourne, VIC, Australia). PDI204 has been demonstrated to be stable *in vitro* over a minimum of 12 months at −20°C.

Before the clinical trial, Good Laboratory Practice (GLP)‐compliant repeat‐dose toxicology studies were done in Sprague–Dawley rats at Syngene (Bangalore, India). PDI204 was administered to the rats either IM (20 mg) or IV (30, 100 and 200 mg/kg) on days 1 and 15. Repeated administration of PDI204 via IM or IV routes was well‐tolerated without adverse effects. The no observed adverse effect level (NOAEL) was 200 mg/kg for IV and 20 mg per rat for IM, which corresponded to the highest doses tested. Human plasma membrane protein cell arrays and tissue cross reactivity studies with PDI204 were conducted. PDI204 was found to be highly specific for SARS‐CoV‐2 spike and had no unwanted cross reactivity to human proteins.

### Clinical trial

A randomised double‐blinded first‐in‐human phase I trial was initiated with the primary objective of evaluating the safety and tolerability of a single IV or IM dose of PDI204 in healthy adult participants (clinicaltrial.gov NCT 06965751, full protocol is available as a Supplementary file 1). Exclusion criteria included COVID‐19 infection or vaccination 120 days prior to PDI204 administration. The trial schema is shown in Supplementary figure 1, with screened healthy adults receiving one of 4 doses of PDI204 (1200 mg IV, 600 mg IV, 200 mg IV or 300 mg IM) or placebo. A staggered dosing schedule was used, with two sentinel participants per dose group (one active, one placebo) studied prior to dosing the remaining six participants (5 active, one placebo). Serial blood and saliva samples were obtained out to 89 days post dosing (Supplementary figure 1). Adverse events were assessed using the Common Terminology for Adverse Events (CTCAE), version 5.0. The trial was approved by the Alfred Hospital Ethics Committee (approval 327/25), and written informed consent was obtained from all participants prior to enrolment in the study.

### Research laboratory evaluation

The concentration of PDI204 in serum was measured with a capture ELISA using an anti‐PDI204 idiotypic antibody (22E11), which was generated from mice immunised with PDI204 and purified from mouse hybridomas. Briefly, diluted serum samples were added to ELISA plates (Nunc) coated with 1 μg/mL of 22E11. Captured PDI204 was detected by secondary mouse anti‐human IgG HRP (ThermoFisher #A‐10648) followed by TMB substrate (Sigma) and read at 450 nm. PDI204 concentration in serum was interpolated using four parameter logistic curve fit with a standard curve. The ELISA was validated according to the International Council for Harmonisation (ICH), Harmonised Guideline “Bioanalytical Method Validation and Study Sample Analysis M10^30^”. The assay had a lower limit of quantification (LLOQ) of 100 ng/mL, a sample dilution range of 1:100 to 1:2000 and met precision, accuracy and selectivity criteria consistent with ICH Guidelines.

To calculate the pharmacokinetic parameters of PDI204 in serum, terminal phase points were identified by visual inspection, selecting 3–5 datapoints from the log‐linear post‐peak decline. Residual area under the curve (AUC) was low, averaging <5% of total AUC, with a maximum of 16%. Individual pharmacokinetic parameters were estimated using non‐compartmental methods. The area under the concentration–time curve from time zero to the last measurable concentration (AUC_0–t_) was calculated using the linear trapezoidal rule. The terminal elimination rate (k_el_) and half‐life were obtained using log‐linear regression on the final 3–5 points of the terminal phase. AUC extrapolated to infinity (AUC_0–inf_) was computed as AUC_0–t_ plus the final measurable concentration divided by k_el_. Clearance (CL) was calculated as dose divided by AUC_0–inf_, and volume of distribution (V_z_) as clearance divided by k_el_.

The concentration of PDI204 in saliva was measured similarly but using the bead‐based multiplex assay. Multiplex beads (Diasorin) were covalently coupled to 22E11 (1 μg/100 μL bead stock) to capture PDI204 from saliva samples. Saliva samples were incubated with the beads at a 1:4 dilution. PE‐conjugated anti‐human IgG1 (Southern Biotech; #9052‐09; 4E3) was used to detect PDI204 in saliva captured by the beads, and concentrations were interpolated from a PDI204 standard curve.

Anti‐drug antibodies (ADA) were assessed by an electrochemiluminescent bridging immunoassay (MSD). Briefly, serum samples were acidified to dissociate pre‐existing PDI204/ADA complexes. Samples were then mixed with biotinylated PDI204 and sulfo‐tag conjugated PDI204 before being added to blocked streptavidin plates. Plates were developed with Gold Read Buffer and analysed on the Meso QuickPlex SQ 120 (MSD plate reader). The positive control used was polyclonal sera from rabbits immunised with PDI204. The assay was validated according to the US FDA Guidance ‘Immunogenicity Testing of Therapeutic Protein Products—Developing and Validating Assays for Anti‐Drug Antibody Detection Guidance for Industry^31^’. The assay had a screening sensitivity of 13 ng/mL and met criteria for precision and selectivity according to the US Guidance.

Spike and nucleocapsid binding IgG and Fc receptor‐engaging antibodies were assessed using a bead‐based multiplex as previously described^32^ (Supplementary figure 7a–f). Neutralising serum antibodies were assessed with a live virus assay as previously described.^33^ Surrogate neutralising activity in saliva was assessed with an ACE2‐RBD inhibition assay as previously described^34^, ^35^ (Supplementary figure 7g).

## Author contributions

**Wen Shi Lee:** Investigation; writing – original draft; writing – review and editing. **Kevin J Selva:** Investigation; writing – review and editing; writing – original draft. **Timothy E Schlub:** Formal analysis; writing – review and editing. **Jennifer Audsley:** Project administration; writing – review and editing; writing – original draft. **Janavi S Rambhatla:** Project administration; writing – review and editing. **Robyn Esterbauer:** Investigation; writing – review and editing. **Jennifer A Juno:** Investigation; writing – review and editing. **Andrew Kelly:** Investigation; writing – review and editing. **Ellie Reilly:** Investigation; writing – review and editing. **Thakshila Amarasena:** Investigation; writing – review and editing. **Sarah Lee:** Investigation; writing – review and editing. **Hyon‐Xhi Tan:** Investigation; writing – review and editing. **Chee Wah Tan:** Resources; writing – review and editing. **Lin‐Fa Wang:** Resources; writing – review and editing. **Christina C Chang:** Investigation; writing – review and editing. **Michael Gerometta:** Investigation; writing – review and editing. **Judith A Scoble:** Investigation; writing – review and editing. **Patrick F James:** Investigation; writing – review and editing. **Arnold Reynaldi:** Formal analysis; writing – review and editing. **Miles P Davenport:** Formal analysis; writing – review and editing. **Kym Hoger:** Investigation; writing – review and editing. **Karen Hughes:** Investigation; writing – review and editing. **Martina L Jones:** Investigation; writing – review and editing. **Ben Hughes:** Investigation; writing – review and editing. **Adam K Wheatley:** Conceptualization; writing – review and editing; funding acquisition; supervision. **Stephen J Kent:** Conceptualization; funding acquisition; writing – review and editing; writing – original draft; supervision.

## Conflict of interest

The authors declare no conflict of interest.

## Supporting information


Supplementary file 1

Supplementary figure 1
Supplementary figure 2
Supplementary figure 3
Supplementary figure 4
Supplementary figure 5
Supplementary figure 6
Supplementary figure 7

Supplementary table 1
Supplementary table 2
Supplementary table 3

## Data Availability

The data that support the findings of this study are available from the corresponding author upon reasonable request.
