# Supplementary material for: Randomised controlled phase I trial of a SARS‐CoV‐2 monoclonal antibody
Source: Clin Transl Immunology. 2026 Nov 4;15(11):e70126. doi: 10.1002/cti2.70126 (PMC13735506; doi:10.1002/cti2.70126)
Supplement: Supplementary file 1 [file CTI2-15-e70126-s001.pdf]

PROTOCOL 7057716
UoM-01-PDI204

A PHASE 1, RANDOMIZED, DOUBLE-BLIND, PLACEBO-CONTROLLED STUDY TO EVALUATE THE SAFETY, TOLERABILITY, AND PHARMACOKINETICS OF SINGLE ASCENDING DOSES OF PDI204 AS INTRAVENOUS INFUSION OR INTRAMUSCULAR INJECTION IN HEALTHY PARTICIPANTS

**Sponsor:**	The University of Melbourne<br>Parkville, Victoria 3010<br>Australia
**Contract Research Organization:**	Syneos Health
**Protocol Version:**	Amendment I
**Date:**	27-JUN-2025

CONFIDENTIAL

This document is strictly confidential. It was developed for The University of Melbourne by Syneos Health. The information in this document may be disclosed only to persons involved in the study, ethics committees, regulatory agencies and study audit personnel. This document is copyrighted in favor of Syneos Health and cannot be reproduced, modified, or adapted, in part or in total, without prior written approval by Syneos Health.

PROTOCOL HISTORICAL FILE

Version	Brief description/summary of changes	Date
Final	Version submitted to the Independent Ethics Committee (IEC).	14-MAY-2025
Amendment I	Please see summary of changes below.	27-JUN-2025

The following changes are brought to the Final Protocol dated 09 MAY 2025:

- **Addition of Unblinded Drug Administrators and Clarification on Blinding Procedures for Drug Administration:**

The Protocol has been updated to allow for the use of unblinded personnels for drug administration in the event of visible or otherwise detectable differences between the active drug and placebo. Also, to ensure the integrity of the study blind, procedures such as participant blindfolding during administration may be implemented when necessary.

Accordingly, the following paragraph has been added to Section 7.3 Randomization and Blinding:

"***If discernible differences between the active IP and placebo are identified, unblinded personnels may be assigned for drug administration. To maintain the study blind, procedures such as participant blindfolding during administration may be implemented when necessary.***"

- **Removal of COVID-19 Testing at Screening and Outpatient Visits, and update in COVID-19 reporting:**

COVID-19 testing at screening and outpatient visits is no longer deemed necessary and its removal will not impact participant safety or eligibility. Also, the Covid-19 reporting by the participants is not mandatory. As such, screening and outpatient COVID-19 testing procedures have been removed from Table 1 Schedule of Assessments, and footnote #15 has been updated from:

"*COVID-19 test will be performed according to clinical site's procedures at screening, check-in (Day -1), and at follow-up visits on Days 3, 5, 7±1, 15±1, 30 ±3, 60 ±3, and at EOS or ET. Any intercurrent COVID- 19 should be tested at home by rapid antigen test (RAT), if participants are symptomatic and results need to be reported to study personnel.*"

To:

"*COVID-19 test will be performed according to clinical site's procedures at check-in (Day -1).* ***Participants experiencing any*** *intercurrent COVID- 19* ***symptoms should be encouraged to self- test*** *at home by rapid antigen test (RAT), and* ***report the results*** *to study personnel.*"

Also, exclusion criteria #16 in Synopsis of Protocol, and Section 6.3 Exclusion Criteria has been updated from:

"*Positive Corona Virus Disease of 2019 (COVID-19) test at screening or admission to the CRU*".

To:

"*Positive Corona Virus Disease of 2019 (COVID-19) test* ***at admission*** *to the CRU*".

- **Resting Period Prior to Vital Signs and 12-Lead ECG Assessments:**

The resting period prior to vital signs has been updated from 5 minutes to 3 minutes in Section 9.1.4 Vital Signs to align with the footnote #8 in Table 1 Schedule of Assessments.

For operational consistency and alignment with vital signs assessments, the resting period prior to 12-lead ECG assessments has also been changed from 5 minutes to 3 minutes. This change is reflected in footnote #10 of Table 1 Schedule of Assessments, and Section 9.1.5 12-lead ECG.

- **Updates to Drug and Alcohol Use Criteria:**

To streamline and harmonize marijuana and other substance use-related eligibility criteria across the protocol, and to align with the intent of Section 8.3 Drugs, nicotine and Alcohol, the following changes have been made:

Inclusion criterion #6 has been updated from:

"Willing to abstain from alcohol, tobacco, and illicit drug use for 48 hours prior to admission to the Clinical Research Unit (CRU) (Day -1) and during the inpatient period."

To:

"Willing to abstain from ***soft or hard drugs (except marijuana) from screening and throughout the study, and from alcohol-based products from 24 hours prior to screening and admission until discharge and from 24 hours prior to each follow-up visit, and from cigarettes or alternative nicotine products during confinement."***

Exclusion criteria #4 has been updated from:

"Positive urine drug screen, or alcohol breath test."

To:

"Positive urine drug screen ***(excluding marijuana)****, or alcohol breath test.* ***Per Investigator's discretion, a single repeat for drug abuse urine test in the event of a false positive is allowed.***"

Exclusion criteria #8 has been updated from:

"*History of drug abuse within 1 year prior to screening or recreational use of soft drugs (such as marijuana) within 1 month or hard drugs (such as cocaine, phencyclidine [PCP], crack, opioid derivatives including heroin, and amphetamine derivatives) within 3 months prior to screening. Per Investigator's discretion, a single repeat for drug abuse urine test in the event of a false positive is allowed.*"

To:

"*History of drug abuse within 1 year prior to screening.*"

These changes are reflected in the Synopsis of Protocol, Section 6.2 Inclusion Criteria, and Section 6.3 Exclusion Criteria.

Also, the first bullet under Section 8.3 Drugs, nicotine and Alcohol, has been updated from:

"*Soft or hard drugs from screening and throughout the study*;"

To:

"*Soft or hard drugs **(except marijuana)** from screening and throughout the study*;"

Additionally, tetrahydrocannabinol (THC) has been removed from Table 4 Clinical Laboratory Assessments, as marijuana use is now permitted during the study.

- **Medical Monitor Reassignment:**

Dr. Jon Rankin has been assigned as the Medical Monitor for this study. Therefore, Section 9.2.6.1 Serious Adverse Event Reporting to the Sponsor, is updated from:

"Any SAE will be reported to the Sponsor (or representative) within 24 hours of learning of the event. The notification must be directed to:
(...) Medical Monitor: David Fuller, MBBS, BPharm (Hons)

Mobile: +61 (0)4 5096 5709

E-mail: david.fuller@syneoshealth.com".

To:

"Any SAE will be reported to the Sponsor (or representative) within 24 hours of learning of the event. The notification must be directed to:

(...) Medical Monitor: ***Jon Rankin, MB BS Grad. Dip. PH SCHP JD(Hons) LLM Grad. Dip. Legal Practice***

Mobile: ***+61 499 222 117***

E-mail: ***jon.rankin@syneoshealth.com****"*

SPONSOR BLIND SIGNATURE PAGE

The University of Melbourne
Parkville, Victoria 3010
Australia

Sponsor's representatives:

______________________________	____________________
Prof. Stephen Kent, MBBS MD	Date
Doherty Institute	
Department of Microbiology and Immunology	
University of Melbourne	

INVESTIGATOR SIGNATURE PAGE

I have carefully read this study protocol and agree that it contains all necessary information required to conduct this study. I agree to conduct the study according to this protocol (including any amendments) and in accordance with the clinical site's Standard Operating Procedures (SOPs), ICH Good Clinical Practice (GCP), all other applicable regulations, and the recommendations laid down in the most recent version of the Declaration of Helsinki.

______________________________ ______________________
Investigator signature **Date**

____
Investigator printed name

____
Name of clinical facility

____
Location of facility
(City, state, country)

TABLE OF CONTENTS

PROTOCOL HISTORICAL FILE 2
SPONSOR BLIND SIGNATURE PAGE 5
INVESTIGATOR SIGNATURE PAGE 6
TABLE OF CONTENTS 7
LIST OF TABLES 9
LIST OF ABBREVIATIONS 10
SYNOPSIS OF PROTOCOL 14
1 INTRODUCTION 23
1.1 BACKGROUND INFORMATION 23
1.2 SUMMARY OF NONCLINICAL STUDIES 23
1.3 SUMMARY OF CLINICAL STUDIES 27
1.4 RATIONALE FOR STUDY CONDUCT 28
1.5 STARTING DOSE AND DOSE ESCALATION SCHEME IN SAD 28
1.6 RATIONALE FOR THE STUDY POPULATION 29
1.7 BENEFIT/RISK ASSESSMENT 30
2 OBJECTIVES 30
3 ENDPOINTS 30
4 STUDY DESIGN 31
4.1 STUDY PROGRESSION 32
5 DOSE ESCALATION CRITERIA 32
5.1 DATA REVIEW BY THE SAFETY REVIEW COMMITTEE 32
5.2 DOSE ESCALATION AND STOPPING RULES 33
6 STUDY POPULATION 35
6.1 NUMBER OF PARTICIPANTS 35
6.2 INCLUSION CRITERIA 35
6.3 EXCLUSION CRITERIA 36
6.4 PARTICIPANT WITHDRAWAL AND REPLACEMENT 38
7 STUDY DRUGS 38
7.1 DRUG SUPPLIES AND ACCOUNTABILITY 38
7.2 IDENTIFICATION OF STUDY DRUGS 39
7.3 RANDOMIZATION AND BLINDING 39
7.4 STUDY DRUG ADMINISTRATION 40
8 STUDY RESTRICTIONS 41
8.1 CONTRACEPTION 41
8.2 CONCOMITANT MEDICATIONS 41
8.3 DRUGS, NICOTINE AND ALCOHOL 42
8.4 DIET 42
8.5 POSTURE AND PHYSICAL ACTIVITY 43
9 STUDY PROCEDURES 43
9.1 SAFETY AND TOLERABILITY ASSESSMENTS 43
9.2 ADVERSE EVENTS 47
9.3 PHARMACOKINETIC ASSESSMENTS 51
9.4 IMMUNOGENICITY ASSESSMENTS 52

9.5 ANALYTICAL METHODS 52
10 STATISTICAL ANALYSES 53
10.1 ANALYSIS POPULATIONS 53
10.2 SAFETY AND TOLERABILITY PARAMETERS 53
10.3 SAFETY AND TOLERABILITY STATISTICAL ANALYSES 53
10.4 PHARMACOKINETIC PARAMETERS 53
10.5 PHARMACOKINETIC STATISTICAL ANALYSES 54
10.6 IMMUNOGENICITY ASSESSMENTS....54
11 DATA COLLECTION 55
12 REGULATORY CONSIDERATIONS AND QUALITY ASSURANCE 55
12.1 IEC APPROVAL OF PROTOCOL AND OTHER STUDY DOCUMENTS 55
12.2 COMPLIANCE 55
12.3 QUALITY ASSURANCE AND MONITORING 55
12.4 CONFIDENTIALITY AND RETENTION OF STUDY RECORDS 56
13 REFERENCES 57

LIST OF TABLES

TABLE 1: SCHEDULE OF ASSESSMENTS 20
TABLE 2: PLANNED PDI204 SINGLE ASCENDING DOSE LEVELS 32
TABLE 3: INFUSION/INJECTION SITE REACTION SCORES 44
TABLE 4: CLINICAL LABORATORY ASSESSMENTS 46
TABLE 5: ADVERSE EVENT RELATIONSHIP CATEGORIES 48
TABLE 6: TIME WINDOWS FOR PK BLOOD SAMPLES 51
TABLE 7: TIME WINDOWS FOR ADAS BLOOD SAMPLES 52
TABLE 8: TIME WINDOWS FOR NABS BLOOD AND SALIVA SAMPLES 52

LIST OF ABBREVIATIONS

ADA	Anti-drug antibody
AE	adverse event
ALT	alanine aminotransferase
ANOVA	analysis of variance
aPTT	activated partial thromboplastin time
AST	aspartate aminotransferase
AUC	area under the concentration-time curve
$AUC_{0\text{-}inf}$	area under the concentration-time curve from time zero to infinity (extrapolated)
$AUC_{0\text{-}t}$	area under the concentration-time curve from time zero until the last observed concentration
$AUC_{0\text{-}\tau}$	area under the concentration-time curve for one dosing interval (τ) at steady-state
BMI	body mass index
BCC	basal cell carcinoma
BP	Blood pressure
BUN	blood urea nitrogen
CDC	Centers for Disease Control and Prevention
CDER	Center for Drug Evaluation and Research
CFR	Code of Federal Regulations
CHMP	Committee for Medicinal Products for Human Use
CI	confidence interval
CK	creatine kinase
Cl	total clearance
Cl/F	apparent clearance
C_{max}	maximal observed concentration
C_{min}	minimal observed concentration
COPD	Chronic Obstructive Pulmonary Disease
COVID-19	Corona Virus Disease of 2019
CRF	case report form
CRO	contract research organization
CRU	Clinical Research Unit
CTCAE	Common Terminology Criteria for Adverse Events
CV	coefficient of variation
CYP	cytochrome P450

DMP	data management plan
ECG	electrocardiogram
eCRF	electronic case report form
eGFR	estimated glomerular filtration rate
EMA	European Medicines Agency
ELISA	Enzyme-linked immunosorbent assay
EOI	end of infusion
EOS	end of study
ET	early termination
FDA	Food and Drug Administration
FIH	first in human
FSH	follicle-stimulating hormone
GCP	Good Clinical Practice
GGT	gamma-glutamyl transferase
GLM	general linear model
GLP	Good Laboratory Practice
GMP	Good Manufacturing Practice
GMT	Geometric mean titer
GSID	genetic sucrase-isomaltase deficiency
HBsAg	hepatitis B surface antigen
HCG	human chorionic gonadotrophin
HCV	hepatitis C virus
HDL-C	high-density lipoprotein cholesterol
HED	human equivalent doses
HEENT	head, eyes, ears, nose, and throat
HIV	human immunodeficiency virus
HPFB	Health Products and Food Branch
HR	heart rate
IB	Investigator’s Brochure
ICF	informed consent form
ICH	International Council for Harmonisation of Technical Requirements for Pharmaceuticals for Human Use
IEC	Independent Ethics Committee
IM	intramuscular
IgG1	Immunoglobulin G1
INR	international normalized ratio

IRB	Institutional Review Board
IV	intravenous
K_{el}	terminal elimination rate constant
LDL-C	low-density lipoprotein cholesterol
LH	luteinizing hormone
LLN	lower limit of normal
mAb	Monoclonal antibody
MAD	multiple ascending dose
MAOI	monoamine oxidase inhibitor
Max	maximum
MCH	mean cell hemoglobin
MCHC	mean cell hemoglobin concentration
MCV	mean cell volume
MDMA	3,4-methylenedioxymethamphetamine
MDRD	modification of diet in renal disease
MedDRA	Medical Dictionary for Regulatory Activities
Min	minimum
mRNA	messenger ribonucleic acid
NAb	neutralizing antibody
NOAEL	no observed adverse effect level
OTC	over-the-counter
PAD	pharmacologically active dose
PCP	phencyclidine
pH	potential of hydrogen
PK	pharmacokinetic(s)
PPE	personal protective equipment
PR	PR interval
PSP	Project Specific Procedure
PT	prothrombin time
PT	preferred term
QA	quality assurance
QC	quality control
QT	QT interval
QTcF	Fridericia’s corrected QT interval
RAT	rapid antigen test
RBC	red blood cell

RBD	Receptor binding domain
RR	Respiratory rate
RT-PCR	reverse transcriptase polymerase chain reaction
SAD	single ascending dose
SAE	serious adverse event
SAP	statistical analysis plan
SARS-CoV-2	severe acute respiratory syndrome coronavirus 2
SAS	statistical analysis system
SCC	squamous cell carcinoma
SD	standard deviation
SOC	System Organ Class
SOP	standard operation procedure
SRC	Safety Review Committee
SUSAR	suspected, unexpected, serious adverse reaction
$T_{½ el}$	terminal elimination half-life
T3	triiodothyronine
T4	thyroxine
TB	tuberculosis
TEAE	treatment-emergent adverse event
TK	Toxicokinetic
T_{max}	time when the maximal concentration is observed
TSH	thyroid-stimulating hormone
ULN	upper limit of normal
URTI	upper respiratory tract infection
V_z	apparent volume of distribution during the terminal phase
V_z/F	apparent volume of distribution
WBC	white blood cell

SYNOPSIS OF PROTOCOL

**Project Number:**	7057716<br>UoM-01-PDI204
**Title:**	A Phase 1, Randomized, Double-Blind, Placebo-Controlled Study to Evaluate the Safety, Tolerability, and Pharmacokinetics of Single Ascending Doses of PDI204 as Intravenous Infusion or Intramuscular Injection in Healthy Participants
**Investigational Product:**	PDI204 injection, provided as 100 mg/mL, will be administered as an intravenous (IV) infusion or intramuscular (IM) injection.<br>The corresponding placebo for PDI204 is 0.9% saline.
**Study Phase and Type:**	Phase 1 - Single Ascending Dose (SAD)
**Objectives:**	Primary objective:<br>• To evaluate the safety and tolerability of a single IV or IM dose of PDI204 in healthy adult participants.<br>Secondary objectives:<br>• To characterize the pharmacokinetic (PK) profile of a single IV or IM dose of PDI204 in serum among healthy adult participants.<br>• To evaluate the presence of anti-drug antibodies (ADAs) in serum after a single IV or IM dose of PDI204 in healthy adult participants.<br>• To explore potential changes in neutralizing antibodies (NAbs) to strains of severe acute respiratory syndrome coronavirus 2 (SARS-CoV-2) in serum and saliva following a single IV or IM dose of PDI204 in healthy adult participants.
**Endpoints:**	Primary endpoint:<br>• Safety and tolerability:<br>o Adverse events (AEs), serious adverse events (SAEs), vital signs measurements (blood pressure, heart rate, respiratory rate, and body temperature), 12-lead electrocardiogram (ECG) recordings, physical examinations, infusion/injection site reactions, and clinical laboratory test results, including hematology, biochemistry, and urinalysis.<br>Secondary endpoints:<br>• PK of PDI204:<br>o The following PK parameters will be calculated:<br>Serum: AUC_{0-t}, AUC_{0-inf}, C_{max}, T_{max}, Residual area, $T_{½ el}$, K_{el}, Cl, V_z, Cl/F, and V_z/F.<br>• Incidence of ADAs.<br>• Serum PK parameters (e.g., C_{max}, AUC) for participants with versus without ADAs.<br>• Changes in SARS-CoV-2 NAb levels in serum and saliva from baseline.
**Study Design:**	This will be a single center, Phase 1, randomized, double-blind, placebo-controlled, sequential SAD study evaluating the safety, tolerability, and PK of PDI204 via a single IV or IM dose in healthy adult participants. The study

	will also assess the incidence and impact of ADAs on PK parameters and evaluate SARS-CoV-2 NAb levels over time.<br><br>The study will consist of a single part with 4 cohorts: 3 sequential cohorts receiving IV administration (Cohorts 1a [200 mg], 2a [600 mg], and 3a [1200 mg]) in an ascending dose manner, and one cohort receiving IM administration (Cohort 2b [300 mg]). Cohort 2b may commence after the safety and tolerability data, as well as available PK data up to at least 96 hours post-dose (Day 5), for a minimum of 6 participants (including sentinels) from Cohort 1a (200 mg – IV) and Cohort 2a (600 mg - IV) are reviewed by the Safety Review Committee (SRC), and may overlap with cohort 3a, if deemed acceptable by the SRC. Each cohort will include approximately 8 participants (6 participants receiving the active and 2 participants receiving the placebo), for a total of approximately 32 participants.<br><br>The study will include a screening visit from Day -28 to Day -2. Eligible participants will be admitted to the clinical site on Day -1 and will be discharged on Day 2 following the completion of all required assessments. Participants will return to the clinical site for follow-up visits on Days 3, 5, 7 (±1), 15 (±1), 30 (±3), 60 (±3), and 90 (±7) days. The total duration of study participation for each participant from screening through the end of study (EOS) is anticipated to be approximately 118 days.<br><br>A staggered dosing schedule will be used for dosing of each cohort and will include 2 sentinel participants (1 active and 1 placebo) dosed initially, and the remaining 6 participants dosed at least 24 hours later in Cohorts 1a, 2a, and 3a (IV administrations); and at least 48 hours later in Cohort 2b (IM administration).<br><br>The planned dose range for IV administration (Cohorts 1a, 2a, and 3a) is anticipated to be from 200 mg to 1200 mg, while a single 300 mg dose is planned for IM administration (Cohort 2b).<br><br>Following completion of each dose level, an SRC will review the safety and tolerability data, as well as available PK data up to at least 96 hours post-dose (Day 5), for a minimum of 6 participants (including sentinels) from the same cohort, in order to make decisions whether to escalate to the next dose level, decrease the next dose level, repeat a dose level, or to not evaluate any additional dose. Additionally, the SRC may extend the duration of the IV infusion (Cohorts 1a, 2a, and 3a) if necessary to improve participant safety or tolerability.
**Study Population:**	It is planned to enroll approximately 32 healthy adult males and female volunteers for participation in this study.
**Inclusion/Exclusion Criteria:**	Participants must meet all of the following criteria to be included in the study:<br>1. Male or female, ≥18 and ≤65 years of age, with BMI >18.5 and <32.0 kg/m^2.<br>2. Healthy as defined by:<br>a. The absence of clinically significant illness and surgery within 4 weeks prior to study drug administration.<br>b. The absence of clinically significant history of neurological, endocrine, cardiovascular, respiratory, hematological, immunological, psychiatric, gastrointestinal, renal, hepatic, and metabolic disease. Fully resolved basal cell carcinoma (BCC) and squamous cell carcinoma (SCC) are acceptable.<br>3. Females of non-childbearing potential must be:<br>a. post-menopausal (spontaneous amenorrhea for at least 12 months prior to dosing) with confirmation by documented FSH levels 40 ≥ mIU/mL; or

	b. surgically sterile (bilateral oophorectomy or hysterectomy) at least 3 months prior to dosing.<br>4. Sexually active females of childbearing potential and non-sterile males must be willing to use an acceptable contraceptive method throughout the study as detailed in section 8.1.<br>5. Male participants must be willing not to donate sperm for 120 days and female participants must be willing not to donate eggs for 120 days after dosing.<br>6. Willing to abstain from soft or hard drugs (except marijuana) from screening and throughout the study, and from alcohol-based products from 24 hours prior to screening and admission until discharge and from 24 hours prior to each follow-up visit, and from cigarettes or alternative nicotine products during confinement.<br>7. Non-tattooed, clear injection site (i.e., absence of dermatologic conditions, such as scarring or rash, that may impact the ability to assess injection site reactions) suitable for IV or IM injection and monitoring in the opinion of the Investigator.<br>8. Able to understand the study procedures and provide signed informed consent to participate in the study.<br>Participants to whom any of the following applies will be excluded from the study:<br>1. Any clinically significant abnormal finding at physical examination.<br>2. Clinically significant abnormal laboratory test results at screening or Day -1, or any positive serology test results for HBsAg, HCV antibody, or HIV antigen and antibody at screening. A single repeat for safety laboratory assessment to confirm initial result and trending is allowed per Investigator's discretion.<br>3. Positive pregnancy test or lactating female participant.<br>4. Positive urine drug screen (excluding marijuana), or alcohol breath test. Per Investigator's discretion, a single repeat for drug abuse urine test in the event of a false positive is allowed.<br>5. History of significant allergic reactions (e.g., anaphylactic reaction, hypersensitivity, angioedema) to any drug.<br>6. Clinically significant ECG abnormalities or vital signs abnormalities (systolic Blood pressure (BP) lower than 90 or over 140 mmHg, diastolic BP lower than 40 or over 90 mmHg, Heart rate (HR) less than 40 or over 100 bpm, or Respiratory rate (RR) less than 10 or over 22 bpm) at screening, or Day -1.<br>7. Any of the following laboratory parameters above the upper limit of normal (ULN) values at screening or Day -1: aspartate aminotransferase (AST), alanine aminotransferase (ALT), direct bilirubin, indirect bilirubin, and total bilirubin. Only abnormal values up to 1.5x ULN may be repeated once for confirmation to below ULN. Isolated elevation in unconjugated bilirubin (e.g., suspected Gilbert's syndrome) is not exclusionary.<br>8. History of drug abuse within 1 year prior to screening.<br>9. History of alcohol abuse within 1 year prior to screening or regular use of alcohol within 6 months prior to screening that exceeds 14 units for women and 21 units for men of alcohol per week (1 unit = 200 mL of beer 5%, 83 mL of wine 12%, or 25 mL of distilled alcohol 40%).

	10. Participants who smoke more than 10 cigarettes per day or the equivalent per week.<br>11. History of rare hereditary sucrose intolerance (e.g., genetic sucrose-isomaltase deficiency (GSID).<br>12. History of a known or suspected respiratory system disorder including, but not limited to, cystic fibrosis, interstitial lung disease, reactive airway disease, emphysema, chronic bronchitis, pulmonary hypertension, Chronic Obstructive Pulmonary Disease (COPD), or asthma (participants with childhood asthma can be included in the study).<br>13. Diagnosis or suspected diagnosis of immunodeficiency or autoimmune diseases, or undergoing immunosuppressive therapy such as anticancer chemotherapy or radiotherapy before the study, or has received systemic corticosteroid treatment (topical corticosteroids are acceptable) within the past 120 days before dosing.<br>14. Poor peripheral venous access for Cohorts 1a, 2a, and 3a.<br>15. Fever (≥ 38.0°C) within 14 days before study drug administration.<br>16. Positive Corona Virus Disease of 2019 (COVID-19) test at admission to the CRU.<br>17. Participants who had close contact (without personal protective equipment (PPE)) as defined by the Centers for Disease Control and Prevention (CDC) in the past 14 days prior to screening, with someone diagnosed with SARS-CoV-2 infection or COVID-19 within 10 days of the close contact. Participants may be rescreened after 14 days provided that they remain asymptomatic.<br>18. Participants who have been infected by COVID-19, within 120 days prior to PDI204 administration.<br>19. Vaccination with COVID-19 vaccine within 120 days prior to administration of PDI204 until EOS or ET.<br>20. Vaccination (other than COVID-19 vaccine) within 30 days prior to administration of PDI204 until 30 days post-dose.<br>21. Known or suspected intolerance or hypersensitivity to any biologic medication or known allergies or clinically significant reactions to human proteins, monoclonal antibodies (mAbs) or antibody fragments, or to any components of the formulation of PDI204 and its excipients used in this study.<br>22. History of bleeding disorders or clotting disorders (e.g., hemophilia, thrombocytopenia) or those with a history of easy bruising or bleeding who may be at a higher risk for hematoma at the injection site.<br>23. Has known active infection with influenza or other non-SARS-CoV-2 respiratory pathogen, confirmed by a diagnostic test.<br>24. Previous infusion-related reaction.<br>25. Use of systemic immunosuppressive or immunomodulatory therapies during the study period.<br>26. Use of medications within the timeframes specified in section 8.2.<br>27. Participation in a clinical research study involving the administration of an investigational or marketed drug (including mAbs) or device within 30 days (or 5 half-lives since last receipt of an investigational drug, whichever is longer) prior to the first dosing, administration of a biological product in the context of a clinical research study within 90 days prior to the first dosing, or

	concomitant participation in an investigational study involving no drug or device administration.<br>28. Donation of serum within 7 days prior to dosing or donation or loss of 500 mL or more of whole blood within 30 days prior to screening.<br>29. Any reason which, in the opinion of the Investigator, would prevent the subject from participating in the study.
**Study Drug Administration:**	Participants in each cohort will receive a single IV or IM dose of PDI204, or placebo at the following planned dose levels: (see table below)
**Study Procedures:**	Safety procedures will be performed, and serum samples for PK analysis and immunogenicity, along with serum and saliva samples for SARS-CoV-2 NAb levels, will be collected at pre-defined times throughout the study as specified in Table 1.<br>Participants will be monitored throughout the study by the clinical staff for AEs and concomitant medication use.
**Statistical Analyses:**	<u>Safety and tolerability</u>:<br>Safety and tolerability of PDI204 will be evaluated through the assessment of AEs (i.e., seriousness, severity [using the current Common Terminology for Adverse Events (CTCAE), version 5.0], relationship to the study drug, outcome, duration, and management), SAEs, vital signs, 12-lead ECGs, clinical laboratory tests, physical examinations, and infusion/injection site reaction assessment.<br>Treatment-emergent adverse events (TEAEs) will be tabulated by treatment. Changes from baseline values in vital signs, 12-lead ECGs, and clinical laboratory tests will be evaluated. Safety and tolerability data will be reported using descriptive statistics. The TEAE rates will be summarized by system organ class (SOC) and preferred term (PT).<br><u>PK</u>:<br>Preliminary PK analyses will be performed after completion of each dose level, without revealing the participant numbers in order to preserve the blind. Summary statistics will be used to describe the PK profile for each dose level.<br>The power model approach will be performed on $AUC_{0\text{-}t}$, $AUC_{0\text{-}inf}$, and C_{max} data to assess the dose-proportionality. Statistical comparisons of dose-normalized $AUC_{0\text{-}t}$, $AUC_{0\text{-}inf}$, and C_{max} will be performed for IV treatments vs IM treatment.<br><u>Anti-drug Antibodies:</u><br>The incidence and prevalence of ADAs will be summarized by dose and visit, using descriptive statistics. The impact of ADAs on the PK of PDI204 will be assessed by comparing PK parameters (e.g., $AUC_{0\text{-}t}$, $AUC_{0\text{-}inf}$, and C_{max}) between ADA-positive and ADA-negative participants. Additionally, potential effects of ADA development on safety and serum levels of PDI204 might be explored.<br><u>SARS-CoV-2 Neutralizing Antibodies (NAbs):</u><br>Descriptive statistics will be used to summarize SARS-CoV-2 NAb titers over time in serum and saliva. Geometric mean titers (GMTs) and corresponding 95%

Cohort	Planned PDI204 Dose Level	Route of Administeration
1a	200 mg	IV
2a	600 mg	IV
2b	300 mg	IM
3a	1200 mg	IV

	confidence intervals will be calculated for each time point. Longitudinal analyses may be conducted to assess the durability of the immune response, and statistical comparisons between dose groups may be performed using an appropriate model (e.g., mixed-effects model or ANOVA).

Table 1: Schedule of Assessments

**Study Stage**	**Screening**	**Baseline**	**Treatment Period**		**Follow-Up Visits[16]**						**EOS**	**ET[17]**
**Day**	**-28 to -2**	**-1**	**1**	**2**	**3**	**5**	**7±1**	**15±1**	**30±3**	**60±3**	**90±7**	**--**
Informed consent	X											
Eligibility check/review	X	X										
Demographic data	X											
Medical, and smoking history	X											
Confinement		X	X									
Randomization[1]		X										
Discharge				X								
Outpatient visits					X	X	X	X	X	X	X	X
**Study drug administration**												
PDI204 or placebo			X									
**Pharmacokinetics**												
Serum samples for PK analysis[2]			X	X	X	X	X	X	X	X	X	X
**Immunogenicity Assessments**												
ADAs[3]			X						X		X	X
Nab sassay (serum)[4]			X	X	X		X	X	X		X	X
Nab sassay (saliva)[5]			X	X	X		X	X	X		X	X
**Safety**												
Physical examination[6]	X	X	X	X	X	X	X	X	X	X	X	X
Body measurements (height, weight, BMI)[7]	X	X							X	X	X	X
Vital signs[8]	X	X	X	X	X	X	X	X	X	X	X	X
Infusion/Injection site assessment[9]			X	X	X	X	X	X	X	X	X	X

Study Stage	Screening	Baseline	Treatment Period		Follow-Up Visits[16]						EOS	ET[17]
**Day**	**-28 to -2**	**-1**	**1**	**2**	**3**	**5**	**7±1**	**15±1**	**30±3**	**60±3**	**90±7**	**--**
12-lead ECG[10]	X	X		X								
Serology tests[11]	X											
Clinical laboratory tests[12]	X	X	X	X	X	X		X	X		X	X
FSH (for post-menopausal females)[13]	X											
Pregnancy tests[14]	X	X					X	X	X	X	X	X
COVID-19 test[15]		X										
Drug, and alcohol screens	X	X										
Monitoring and recording of AEs and prior/concomitant medication use	←	―	―	―	―	―	―	―	―	―	―	→

Abbreviations: ADA = Anti-drug antibody; AE = adverse event; BMI = body mass index; COVID-19 = Corona Virus Disease of 2019; ECG = electrocardiogram; EOS= end of study; ET = early termination; FSH = follicle-stimulating hormone; NAb = neutralizing antibody; PK = pharmacokinetic.

1 May also occur before dosing on Day 1 .

2 A total of 13 serum samples will be collected for PK analysis: on Day 1 at pre-dose/pre-infusion, and 30 minutes post-dose/post end of infusion (EOI), and at 4, 8, 12, and 24 (Day 2-prior to discharge) hours post-dose/post-EOI. Further samples to be collected at follow-up visits at 48 (Day 3), 96 (Day 5), 144 (Day 7), 336 (Day 15), 696 (Day 30), and 1416 (Day 60) hours post-dose/post-EOI, and at EOS or ET. Refer to Table 6 for more details.

3 A total of 3 serum samples will be collected for ADAs analysis: on Day 1 at pre-dose (within 60 minutes), and on Day 30±3, and at EOS or ET. Refer to Table 7 for more details.

4 A total of 7 serum samples will be collected for NAbs analysis: on Day 1 at pre-dose (within 60 minutes), Day 2, Day 3, Day 7 ±1, Day 15 ±1, Day 30±3, and at EOS or ET. Refer to Table 8 for more details.

5 A total of 7 saliva samples will be collected for NAbs analysis: on Day 1 at pre-dose (within 60 minutes), Day 2, Day 3, Day 7 ±1, Day 15 ±1, Day 30±3, and at EOS or ET. Refer to Table 8 for more details.

6 A complete physical examination will be performed at screening. A brief physical examination will be performed on Day -1, pre-dose on Day 1 (within 60 minutes), and on Day 2 (before discharge), and at follow-up visits on Days 3, 5, 7±1, 15±1, 30±3, 60±3, and at EOS or ET.

7 Height, weight, and BMI will be measured at screening. Body weight only will be measured on Day -1, and at follow-up visits on Days 30±3, and 60±3, and at EOS or ET.

8 Blood pressure, heart rate, respiration rate, and body temperature will be measured after 3 minutes rest in a supine position at screening, on Day -1, on Day 1 at pre-dose (within 60 minutes), and 2, and 4 hours post-dose/post-EOI (±15 minutes), and 8, 12, and 24 (Day 2-before discharge) hours post-dose/post-EOI (±60 minutes), and at follow-up visits on Days 3, 5, 7±1, 15±1, 30±3, 60±3, and at EOS or ET.

9 Infusion site evaluation for Cohorts 1a, 2a, and 3a and injection site evaluation for Cohort 2b will be performed on Day 1 at pre-dose (within 60 minutes), and 2, 4, 12, and 24 (Day 2) hours post-dose/post-EOI (±15 minutes), and at follow-up visits on Days 3, 5, 7±1, 15±1, 30±3, 60±3, and at EOS or ET. Refer to Section 9.1.3 for infusion/injection site reaction scores.

10 12-lead ECG will be measured at screening, on Day -1, and on Day 2 (24 hours post-dose/post-EOI) (±60 minutes). Single 12-lead ECG will be recorded after the participants have been resting for at least 3 minutes in a supine position

11 Serology tests include HBsAg, HCV antibody, and HIV antigen and antibody.

12 Standard biochemistry, coagulation, hematology, and urinalysis tests will be performed under fasting conditions at screening, on Day -1, on Day 1 at pre-dose/pre-infusion (within 60 minutes), and on Day 2 (before discharge), and at follow-up visits on Days 3, 5, 15±1, 30±3, and at EOS or ET.

13 FSH levels will be measured at screening to confirm the post-menopausal status.

14 Women of childbearing potential only: A serum pregnancy test will be performed at screening. Urine pregnancy test will be performed on Day -1, and at follow-up visits on Days 7±1, 15±1, 30 ±3, 60 ±3, and at EOS or ET.

15 COVID-19 test will be performed according to clinical site's procedures at check-in (Day -1). Participants experiencing any intercurrent COVID- 19 symptoms should be encouraged to self-test at home by rapid antigen test (RAT), and report the results to study personnel.

16 There will be outpatient visits on Days 3, 5, 7±1, 15±1, 30±3, and 60±3, and at EOS or ET.

17 In case of ET, all ET procedures will be performed as soon as possible.

1 INTRODUCTION

1.1 Background Information

Corona Virus Disease of 2019 (COVID-19) is caused by severe acute respiratory syndrome coronavirus 2 (SARS-CoV-2) infection, a global pandemic virus that emerged in late 2019/early 2020. SARS-CoV-2 most commonly causes a mild to moderate influenza-like respiratory illness. However, vulnerable groups including the elderly and immunosuppressed are susceptible to severe COVID-19.[1]

Vaccines and antiviral medications were quickly developed to mitigate the severity of SARS- CoV-2 infection. The widespread use of messenger ribonucleic acid (mRNA) and other vaccines have reduced diseases severity. Neutralizing antibodies were identified as a strong correlate of protection induced by vaccination. However, the changing variants of SARS-CoV-2 strains has meant that vaccine efficacy can be limited against newer strains. Vaccines are currently being regularly updated with newer strains although the rapid pace of change has meant that vaccines are not ideal matches with circulating strains. Further, immunity induced by vaccination wanes over time leaving people vulnerable to re-infection.[1]

Antiviral medications such as Paxlovid and Molnupiravir are available to reduce virus levels and mitigate disease severity. However, these medications must be prescribed early in the illness, or they are less effective. Further, they have not proven effective to prevent illness. There is also concern that SARS-CoV-2 will mutate to become resistant to currently available antiviral medications. There is an urgent need for additional treatments for COVID-19 and therapies to prevent COVID-19, particularly for vulnerable groups.[1]

PDI204 is a fully human, immunoglobulin G1 (IgG1) monoclonal antibody (mAb) directed against the SARS-CoV-2 spike protein. It is being developed by the University of Melbourne for the following indications:

1) The prevention of SARS-CoV-2 infection in adults at high risk of developing severe COVID-19;
2) The reduction in severity of COVID-19 breakthrough infection in adults at risk of severe COVID-19.

For more information, refer to Investigator's Brochure (IB).[1]

1.2 Summary of Nonclinical Studies

To support clinical development of PDI204, the University of Melbourne has conducted the following nonclinical studies.[1]

1.2.1 Nonclinical Pharmacology

1.2.1.1 Primary Pharmacology

1.2.1.1.1 Assessment of the Binding Profile of PDI-204 Antibody Using a Human Plasma Membrane Protein Cell Array (Non-GLP)

The objective of this study was to screen for specific off-target binding interactions of the test PDI204 antibody, primarily against the SARS-CoV-2 spike protein.[1]

Pre-screens were performed to assess background binding of PDI204 to untransfected HEK293 cells and to cells transfected with human CD20 or epidermal growth factor receptor (EGFR). Cells were exposed to various concentrations of PDI204 (from 0.25 to 20 µg/mL), or rituximab (1 µg/mL), a biosimilar positive control mAb, or a phosphate-buffered saline (PBS) control solution.[1]

Among 37 screened protein targets, 28 showed binding, although these were deemed non- specific interactions.[1]

Only the added SARS-CoV-2 spike protein showed a strong, specific interaction with the test antibody, confirming the binding specificity of PDI204. Further confirmation was provided by the live-cell screen, which indicated no significant specific interactions.[1]

For more information, refer to IB.[1]

1.2.1.1.2 Tissue Cross Reactivity Study with FITC-Labelled PDI204 in Normal Human Tissues (GLP)

The potential cross-reactivity of FITC-labelled PDI204 was evaluated using the human IgG1 kappa mAb directed against SARS-CoV-2 spike protein, in cryosections from a full panel of normal human tissues.[1]

Two concentrations of the PDI204-FITC test article (0.25 µg/mL and 2 µg/mL) were applied to the cryosections. A human IgG1κ mAb (HuIgG1-FITC), which has a different antigenic specificity from that of PDI204-FITC, was designated as a cross-reactivity control article, along with general assay controls.[1]

PDI204-FITC showed moderate to strong cytoplasmic staining of spindle cells in the peripheral nerve in 2 out of approximately 35 nerve fascicles. All other tissues showed no staining for both PDI204-FITC and HuIgG1-FITC. The cytoplasmic binding of mAbs was considered to have little to no toxicological relevance due to their general inability to access the cytoplasm *in vivo*.[1]

For more information, refer to IB.[1]

1.2.1.1.3 Preliminary Efficacy Assessment in Mice (Non-GLP)

The efficacy of PDI204 was tested in a transgenic human ACE-2 mice against SARS-CoV-2 Delta variant, in both therapeutic and prophylactic contexts.[1]

In the therapeutic study, mice (n=5 per group) infected with the Delta variant received a single intraperitoneal dose of PDI204 (1 or 5 mg/kg) or 5 mg/kg of an isotype control, administered 24 hours post-infection.[1]

On Day 4 post-infection, lung viral loads was significantly reduced in PDI204 groups relative to negative control in a dose-dependent manner, whereas viral load in nasal turbinates was completely suppressed. Mortality was also significantly reduced with only transient weight loss over 15 days.[1]

In the prophylactic study, mice (n=5 per group) received PDI204 (1 or 5 mg/kg) or an isotype control (5 mg/kg) via intraperitoneal route 24 hours pre-infection intranasally with SARS-CoV-2 Omicron BA.1 variant. By Day 4 post-infection, lung viral loads were completely suppressed in all PDI204 treated groups, and viral loads in nasal turbinates were significantly reduced in a dose-dependent manner.[1]

These results support the preliminary therapeutic and prophylactic efficacy of PDI204 *in vivo*.[1]

For more information, refer to IB.[1]

1.2.2 Pharmacokinetics and Product Metabolism in Animals

1.2.2.1 Pharmacokinetic Profiles of PDI204 Following Single Intravenous Administrations in C57/BL6 Mice

Female C57/BL6 mice (n=5 per group) were injected intravenously with 1 µg or 5 µg of PDI204 corresponding to doses of approximately 0.05 mg/kg and 0.25 mg/kg. Serological concentrations of PDI204 were assessed over time using a murine enzyme-linked immunosorbent assay (ELISA)-based PK assay.[1]

Serological detection of PDI204 was found to be extending >50 days in animals receiving 1 µg, and >70 days in animals receiving 5 µg.[1]

For more information, refer to IB.[1]

1.2.3 PDI204 Monoclonal Antibody: 4-Weeks (2 Doses) Toxicity Study by IV and IM Routes with 14-day Recovery in Sprague Dawley Rats / Study Number U-23340 (GLP)

This study evaluated the potential systemic toxicity, target organs, toxicokinetic (TK) profile, and reversibility of toxicity of PDI204 by IV (slow bolus) and IM routes in Sprague-Dawley rats.[1]

In the toxicity study, animals were administered PDI204 on Days 1 and 15, through IM route at a single maximum permissible dose of 20 mg/rat, or IV route at single doses of 30, 100, and 200 mg/kg (total of 2 injections for every group). Vehicle control-treated groups (0 mg/kg, normal saline) were included for both IV and IM routes. TK animals were to receive a single dose of PDI204 or control on Day 1 only.[1]

The mean T_{max} values for PDI204 following IM administration was 24 and 48 hours on Day 1 at 20 mg/rat in male and female rats, respectively. Mean T_{max} values regarding all dose levels of IV administrations were between 0.5 to 2.0 hours, and 0.5 and 6.0 hours for males and females, respectively. Quantifiable concentrations of PDI204 were detected up to the last collection timepoint (336 hours) across all dose levels in both sexes for each route of administration.[1]

Also, terminal half-life ($T_{½}$) was 107 hours (4 days) in males and 191 hours (7 days) in females via IM administration at 20 mg/rat, respectively. For IV dose administrations, $T_{½}$ values were 278 hours (11 days), 264 hours (11 days), and 170 hours (7 days) in males and 350 hours (14 days), 279 hours (11 days) and 363 hours (15 days) in females for IV administrations at 30, 100, and 200 mg/kg.[1] Overall, increases in IV dose from 30 to 200 mg/kg (6.7-fold) resulted in dose-proportional increases in both $AUC_{0\text{-}336h}$ and C_{max} (7.3-fold each) in both sexes.

Following IM administration, sex-related differences in exposure were approximately 2.4-fold. As for IV administrations, sex differences in C_{max} and $AUC_{0\text{-}336h}$ were around 1 at all dose levels, suggesting no sex differences.[1]

For more information, refer to IB.[1]

1.2.4 Nonclinical Toxicology

1.2.4.1 Repeat-Dose Toxicity Study in Rats

1.2.4.1.1 PDI204 Monoclonal Antibody: 4-Weeks (2 Doses) Toxicity Study by IV and IM Routes with 14-day Recovery in Sprague Dawley Rats / Study Number U-23340

The potential systemic toxicity, target organs, TK profile and reversibility of toxicity of PDI204 were evaluated in Sprague-Dawley rats via IV (slow bolus) and IM routes.[1]

The animals were divided into 6 main groups (10 rats/sex/group), and 6 recovery groups (5 rats/sex/group). On Days 1 and 15, animals received a single dose of PDI204 through IM route (20 mg/rat), or IV route (30, 100, and 200 mg/kg), or corresponding IM or IV controls. The site of injection was the lateral thigh muscle for IM and lateral tail vein for IV administrations.[1]

No mortality or clinical signs of toxicity were observed. There were no test item-related changes in mean bodyweight, mean bodyweight change, or food consumption compared to control groups.[1]

No test item-related changes were observed in hematology, coagulation, or urinalysis parameters at both terminal (Day 16) and recovery (Day 30) timepoints. However, at terminal sacrifice and recovery sacrifice, statistically significant increases in globulin levels were noted in females, and decreases in albumin-to-globulin ratio were noted in both sexes following IM administration of PDI204 .

Increases in total protein and globulin levels, along with decreases in albumin-to-globulin ratios, were observed in males at doses ≥ 100 mg/kg administered via the IV route. Similar changes were noted in females, with increased globulin at 200 mg/kg and reduced albumin-to-globulin ratios at ≥100 mg/kg. Triglyceride levels increased at 200 mg/kg in both sexes. At recovery sacrifice, the decreased albumin-to-globulin ratio remained statistically significant in females at 200 mg/kg. All other changes resolved by the end of the recovery period and were considered test article-related, but non-adverse.[1]

At terminal sacrifice and recovery sacrifice, no PDI204-related changes were noted in necropsy findings or organ weights across all dose levels and administration routes. A red focus at the IM injection site was noted in one female in the test item group and was considered procedure-related change.[1]

No test item-related microscopic changes were noted in animals administered with PDI204 via IV route at terminal sacrifice. However, in rats administered PDI204 (20 mg/rat) intramuscularly, liver changes such as minimal hypertrophy of sinusoidal cells and minimal to mild increased in mitosis of hepatocytes were observed in both sexes. These findings persisted at the end of the recovery period, with increased mitosis still present in males. In the lungs, interstitial/alveolar infiltration of mononuclear/mixed cells was observed in both vehicle control and PDI204 groups administered via IV, indicating it was not test article-specific.[1]

Microscopic findings at the IV injection site (tail vein) included perivascular mixed cell infiltration, hemorrhage, and fibrosis in both vehicle control and PDI204 groups, along with epidermal crust in a few rats. At the IM injection site (lateral thigh), hemorrhage and/or mononuclear/mixed cell infiltration were observed in both vehicle control- and PDI204-administered groups. These changes were considered procedure- related. All other microscopic changes were deemed incidental or spontaneous and unrelated to PDI204.[1]

Repeated administration of PDI204 via IV or IM routes on Days 1 and 15 in Sprague-Dawley rats was well tolerated without adverse effects. The no observed adverse effect level (NOAEL) was 200 mg/kg for IV and 20 mg/rat for IM, which corresponded to the highest doses tested.

For more information, refer to IB.[1]

1.3 Summary of Clinical Studies

PDI204 has not been tested in clinical trials.

1.3.1 Safety and Tolerability in Humans

To date, no clinical data is available on the safety of SARS-CoV-2-neutralising PDI204 mAb. However, a comparable IV-administered SARS-CoV-2-neutralising mAb, pemibivart (Pemgarda™), has been evaluated in an ongoing Phase 3 clinical trial. IV administration of pemibivart in over 600 participants has demonstrated an acceptable safety and tolerability profile, with most adverse events being mild to moderate.[1]

1.3.2 Efficacy in Humans

To date, no clinical efficacy studies have been conducted with the novel SARS-CoV-2 neutralizing PDI204 mAb. However, efficacy data from pemivibart (Pemgarda™), a comparable mAb, support its potential benefit. In a Phase 3 trial, pemivibart demonstrated a reduction in the incidence of reverse transcriptase polymerase chain reaction (RT-PCR)-confirmed symptomatic COVID-19 and COVID-related hospitalizations and deaths in a high-risk population. While differences in assay methodologies and limited comparability in EC50 values restrict direct translation, available pre-clinical data are considered supportive of the potential clinical benefit of PDI204.[1]

1.3.3 Pharmacokinetics and Product Metabolism in Humans

To date, clinical studies to demonstrate absorption, distribution, metabolism and excretion of PDI204 have not been performed. This Phase 1 first in human (FIH) clinical trial aims to determine the serum PK profiles of PDI204. [1]

1.4 Rationale for Study Conduct

As an initial trial in the clinical development of PDI204, The University of Melbourne proposes a Phase 1, randomized, double-blind, placebo-controlled study to evaluate the safety, tolerability, PK, ADAs, and SARS-CoV-2 NAbs response following the administration of a single IV or IM dose in healthy adult participants.

The SAD doses of 200, 600, and 1200 for IV administration and 300 mg for IM administration were selected based on nonclinical pharmacology, PK, and toxicology studies in rats as presented above (see IB for detailed overview).[1]

Using the NOAELs obtained in rats, the human equivalent doses (HEDs) were calculated as presented in the section below.

1.5 Starting Dose and Dose Escalation Scheme in SAD

1.5.1 Starting Dose Selection

The calculation of the starting dose for the SAD part of this study was done based on the methodology described in the FDA Guidance on *Estimating the Maximum Safe Starting Dose in Initial Clinical Trials for Therapeutics in Adult Healthy Volunteers*[2] and considering the recommendations laid down in the EMA *Guideline on strategies to identify and mitigate risks for first-in-human and early clinical trials with investigational medicinal products*.[3]

The starting dose of 200 mg for IV administeration was selected based on the totality of the data from the nonclinical pharmacology, PK, and toxicology studies.

The NOAEL observed was 200 mg/kg for IV administration in rats. The NOAEL was converted into Human Equivalent Doses (HEDs) by division using a conversion factor of 6.2 and assuming that doses scale 1:1 between animal models and humans, when normalized to body surface area. The HED obtained was approximately 32.26 mg/kg corresponding to approximately 1936 mg for an average human body weight of 60 kg. The IV starting dose of 200 mg provides a dose-based safety margin of approximately 10-fold as compared to the HED calculated from the IV NOAEL in rats. Furthermore, the toxicology study included two administrations, on Days 1 and 15, whereas the clinical study involves a single-dose administration. The difference introduces an inherent additional safety which further supports the selected starting dose.

The only dose for IM administration (Cohort 2b) is 300 mg. This 300 mg IM dose will be started once the safety and tolerability data, as well as available PK data up to at least 96 hours post-dose (Day 5), from Cohorts 1a (200 mg – IV), and 2a (600 mg – IV) are available and reviewed by the SRC for a minimum of 6 participants (including sentinels) from each of the cohorts, and may be administered in parallel with Cohort 3a (1200 mg-IV). It is not expected that the exposures achieved in Cohort 2b (300 mg IM) will reach those observed in Cohort 2a (600 mg IV).

1.5.2 Dose Escalation Scheme

The proposed escalation scheme for this SAD study consists of increments by factors of 3 and 2 fold. The planned dose range of 200 mg to 1200 mg was selected to minimize the risk to the participants while ensuring the evaluation of an exposure range that encompasses and exceeds the systemic exposure where pharmacological effects are anticipated. Based on the favorable safety profile in the toxicology studies, a dose escalation scheme of 2- to 3-fold is supported and not expected to cause any safety concerns in healthy adult participants.

Throughout the study, dose levels may be adjusted within the planned dose range based on available safety, tolerability, and PK data from completed cohorts. If a dose level changes from plan, the revised dose will remain within the planned dose range and will not exceed the maximum dose currently outlined in the protocol.

The maximum planned IV dose in this SAD study is 1200 mg and this dose is lower than the HED obtained from the IV NOAEL in rats (approximately 1936 mg).

1.5.3 Rationale for the Selection of Doses Based on Other Approved mAbs

Prior mAbs targeting SARS-CoV-2 have been administered IV at high doses up to 8,000 mg. Pemivibart (Pemgarda™) is currently available as a 4,500 mg dose IV. Previous analyses of aggregated data from randomized controlled trials of SARS-CoV-2 mAbs used for modeling dose-response relationships of mAb for prophylaxis suggest that such higher doses are sometimes in excess of what is required to achieve adequate neutralization titer for COVID-19 protection.[1]

Relative to the planned dose for PDI204, other SARS-CoV-2 mAbs administered IM have been at a dose level of 300 mg, like tixagevimab/cilgavimab (Evusheld™). Moreover, further concentrating mAb formulations beyond 100 mg/mL remains challenging due to unfavorable physicochemical properties during manufacture (e.g. protein aggregation, solubility thresholds, decreased stability). The three IV dose levels aim to demonstrate the safety profile, while establishing an adequate PK profile, therefore providing a margin of safety.[1]

1.6 Rationale for the Study Population

A healthy volunteer population has been selected for the study because healthy adult participants with no concomitant diseases and using no concomitant medications represent a homogenous population allowing for proper evaluation of the safety, tolerability, and PK profile of a drug without confounding factors.

No reproductive studies in animals have been conducted with PDI204 so far. Since the risks to a developing human fetus or a nursing infant following exposure to PDI204 are unknown at this time, only male and non-pregnant, non-lactating female participants will be enrolled in this study. Sexually active female participants of childbearing potential and non-surgically sterile male participants with female partners of childbearing potential will be required to use a double contraceptive method. Pregnancy tests will also be performed at different times over the course of the study for female participants.

1.7 Benefit/Risk Assessment

The nonclinical safety studies support the administration of PDI204 in humans. In nonclinical studies, PDI204 was well-tolerated and without evidence of significant toxicology in animal species at the proposed human doses.

The inclusion and exclusion criteria have been chosen to select participants who are known to be free from any significant illness, history of autoimmune diseases, and from any condition that could impact their safety or interfere with meeting the study objectives. A placebo is included to enable characterization of AEs by serving as a control. PDI204 is not anticipated to provide any benefit to the study participants, and therefore the use of placebo will not deprive participants of any potential benefit from PDI204. There is no anticipated therapeutic benefit for healthy adult participants.

2 OBJECTIVES

Primary objective:

- To evaluate the safety and tolerability of a single IV or IM dose of PDI204 in healthy adult participants.

Secondary objectives:

- To characterize the PK profile of a single IV or IM dose of PDI204 in serum among healthy adult participants.
- To evaluate the presence of ADAs in serum after a single IV or IM dose of PDI204 in healthy adult participants.
- To explore potential changes in NAbs to strains of SARS-CoV-2 in serum and saliva following a single IV or IM dose of PDI204 in healthy adult participants.

3 ENDPOINTS

Primary endpoints:

- Safety and tolerability:
    - AEs, SAEs, vital signs measurements (blood pressure, heart rate, respiratory rate, and body temperature), 12-lead ECG recordings, physical examinations, infusion/injection site reactions, and clinical laboratory test results, including hematology, biochemistry, and urinalysis.

Secondary endpoints:

- PK of PDI204:
    - The following PK parameters will be calculated:

      Serum: AUC_{0-t}, AUC_{0-inf}, C_{max}, T_{max}, Residual area, $T_{½ el}$, K_{el}, Cl, V_z, Cl/F, and V_z/F.

- Incidence of ADAs.
- Serum PK parameters (e.g., C_{max}, AUC) for participants with versus without ADAs.
- Changes in SARS-CoV-2 NAb levels in serum and saliva from baseline.

4 STUDY DESIGN

This will be a single center, Phase 1, randomized, double-blind, placebo-controlled, sequential SAD study evaluating the safety, tolerability, and PK of PDI204 via a single IV or IM dose in healthy adult participants. The study will also assess the incidence and impact of ADAs on PK parameters and evaluate SARS-CoV-2 NAb levels over time.

The study will consist of a single part with 4 cohorts: 3 sequential cohorts receiving IV administration (Cohorts 1a [200 mg], 2a [600 mg], and 3a [1200 mg]) in an ascending dose manner, and one cohort receiving IM administration (Cohort 2b [300 mg]). Cohort 2b may commence after the safety and tolerability data, as well as available PK data up to at least 96 hours post-dose (Day 5), for a minimum of 6 participants (including sentinels) from Cohorts 1a (200 mg - IV), and 2a (600 mg - IV) are reviewed by the SRC, and may overlap with Cohort 3a, if deemed acceptable by the SRC. Each cohort will include approximately 8 participants (6 participants receiving the active and 2 participants receiving the placebo), for a total of approximately 32 participants.

The study will include a screening visit from Day -28 to Day -2. Eligible participants will be admitted to the clinical site on Day -1 and will be discharged on Day 2 following the completion of all required assessments. Participants will return to the clinical site for follow-up visits on Days 3, 5, 7 (±1), 15 (±1), 30 (±3), 60 (±3), and 90 (±7).

The total duration of study participation for each participant from screening through the EOS is anticipated to be approximately 118 days.

A staggered dosing schedule will be used for dosing of each cohort and will include 2 sentinel participants (1 active and 1 placebo) dosed initially, and the remaining 6 participants dosed at least 24 hours later in Cohorts 1a, 2a, and 3a (IV administrations); and at least 48 hours later in Cohort 2b (IM administration).

The planned dose range for IV administration (Cohorts 1a, 2a, and 3a) is anticipated to be from 200 to 1200 mg, while a single 300 mg dose is planned for IM administration (Cohort 2b), as outlined in Table 2.

Following completion of each dose level, an SRC will review the safety and tolerability data, as well as available PK data up to at least 96 hours post-dose (Day 5), for a minimum of 6 participants (including sentinels) from the same cohort before the start of dosing the following cohorts. The SRC will make decisions whether to escalate to the next dose level, decrease the next dose level, repeat a dose level, or to not evaluate any additional dose. Additionally, the SRC may extend the duration of the IV infusion (Cohorts 1a, 2a, and 3a) if necessary to improve participant safety or tolerability.

Table 2: Planned PDI204 Single Ascending Dose Levels

Cohort	Planned PDI204 Dose Level	Route of Administeration
1a	200 mg	IV
2a	600 mg	IV
2b	300 mg	IM
3a	1200 mg	IV

4.1 Study Progression

The sentinels and the remaining participants in a new cohort will not be dosed until the clinical portion of the preceding dose level has been completed, appropriate data has been reviewed by the SRC, and authorization to proceed with the next dose level or next study part has been given by the SRC, as described in the next section.

Adjustment to the currently outlined dose levels and dosing regimen may be implemented by the SRC based on data from completed dose levels. However, the total daily dose to be administered in a given cohort will not exceed the maximum dose currently outlined in the protocol. Additionally, the SRC may extend the duration of the IV infusion (Cohorts 1a, 2a, and 3a) if deemed beneficial for safety or tolerability of study participants.

5 DOSE ESCALATION CRITERIA

5.1 Data Review by the Safety Review Committee

The SRC will be responsible for the assessment of safety, tolerability, and PK data for each dose level and to make decisions with regards to study progression. The SRC will be composed of at least the Investigator, one medically qualified Sponsor representative or delegate and an Independent Medical Monitor.

An SRC meeting will be held after safety and tolerability data, as well as available PK data up to at least 96 hours post-dose, for a aminimum of 6 participants (including sentinels) from the same cohort before the start of dosing of the following cohorts.

The SRC will consider the data on a cohort basis, but also on the basis of cumulative information across cohorts as the study progresses.

The potential decisions of the SRC are:

- Escalate to the next planned dose level;
- Continue the study with a more conservative approach, such as decreasing the next dose level, repeating a dose level, or exploring an alternative dosing regimen (not exceeding the total daily dose planned in the protocol for that given cohort);
- Suspend dose escalation until further review of study data can be made, allowing the SRC to determine whether predefined stopping rules have been met. Dose escalation may resume once the SRC concludes that no stopping rules have been met;

- Stop dose escalation;
- Terminate the study.

The SRC will conduct a blinded review of the data; however the blind may be broken for individual participants if judged necessary, prior to making a decision on dose escalation. Dose escalation may occur only following mutual agreement between the SRC members. The SRC may recommend to modify the PK sample collection schedule based on emerging data, including shortening or lengthening the total collection interval by up to 24 hours, and may add up to two additional PK samples, if necessary. Decisions taken by the SRC will be documented and submitted to the Independent Ethics Committee (IEC) overseeing the study, if required, along with supportive data.

5.2 Dose Escalation and Stopping Rules

The following sections define stopping rules resulting in an immediate stop to dosing. Each section specifies whether the stop involves a final end of dosing or a temporary halt.

5.2.1 Individual Participant Stopping Rules (Cohorts 1a, 2a, and 3a)

IV infusion in Cohorts 1a, 2a, and 3a will be permanently stopped for any individual participant experiencing any of the following:

- SAE, regardless of causality to the study drug.
- Severe AE considered at least possibly related to the study drug.
- Hypersensitivity or anaphylactic reaction following study drug administration.
- Medical condition that is judged by the Investigator as to jeopardize the participant’s safety if he/she continues to receive the study drug.

5.2.2 Cohort Stopping Rules

5.2.2.1 Stopping Rules After Sentinel Dosing

The following events occurring in one sentinel participant will result in a temporary halt of dosing within that cohort:

- The occurrence of a SAE considered at least possibly related to the study drug.
- The occurrence of a CTCAE grade 4 considered at least possibly related to the study drug.

If any of the above stopping rules is met, the remaining participants of the cohort will not be dosed until the SRC reviews details of the event and/or additional data. If, after further review of the data, the SRC determines that no stopping rules have been met (e.g., AE determined to be unrelated to the study drug), then dosing of the remaining participants of the cohort may proceed.

5.2.3 Stopping Rules for Dose Escalation (Cohorts 1a, 2a, and 3a)

Dose escalation in Cohorts with IV administration (1a, 2a, and 3a) will be temporarily halted if the Investigator suspects that any of the stopping rules described in 5.2.3.1 and 5.2.3.2 is met. Dosing in Cohort 2b (300 mg IM) will not start if any of the following stopping rules is met in Cohorts 1a (200 mg IV), and 2a (600 mg IV). Continuation or initiation of cohorts at lower dose levels will be evaluated on a case-by-case basis. If, after further review of the study data, it is determined by the SRC that no stopping rules have been met (e.g., AE determined to be unrelated to the study drug), then dose escalation may resume. However, if the SRC confirms that a stopping rule has been met, dose escalation will be stopped definitively.

5.2.3.1 Events Within One Dose Level

Dose escalation will be halted if any of the events described below occur within one dose level:

- The occurrence of a SAE considered at least possibly related to the study drug in one participant.
- The occurrence of CTCAE grade 4 considered at least possibly related to the study drug in 2 participants and independent of within or not within the same system organ class (SOC).
- Clinically significant abnormalities of the same character, at least of CTCAE grade 3, and considered at least possibly related to the study drug in 2 participants, with regards to:
  - the physical examination;
  - ECGs;
  - vital signs; or
  - clinical laboratory parameters.

5.2.3.2 Cumulative Study Events

In addition to the events listed in 5.2.3.1, any of the events described below occurring at different dose levels administered throughout the study (i.e., cumulative events) will also result in a temporary halt of dose escalation:

- The occurrence of CTCAE grade 4 of the same character considered at least possibly related to the study drug in 2 participants.
- Clinically significant abnormalities of the same character, at least of CTCAE grade 3, and considered at least possibly related to the study drug in 3 participants, with regards to:
  - the physical examination;
  - ECGs;
  - vital signs; or
  - clinical laboratory parameters.
- Determination by the SRC that a pattern of AEs precludes further dose escalation, even if no other stopping rules have been met.

5.2.4 Study Stopping Rules

The study will be terminated if any of the following criteria are met:

- The occurrence of SAEs considered at least possibly related to the study drug in 2 participants and independent of within or not within the same SOC.
- Determination by the SRC that a pattern of AEs precludes any further dosing.

6 STUDY POPULATION

6.1 Number of Participants

It is planned to enroll approximately 32 male or female volunteers for participation in this study.

The study will consist of 4 cohorts, each including approximately 8 participants (6 participants receiving the active and 2 participants receiving the placebo), for a total of approximately 32 participants.

The sample size of this study is not determined based on statistical calculations. A sample size of 8 participants per cohort randomized in a 3:1 ratio to the active versus placebo represents a typical panel for a SAD study and is judged adequate to achieve the study objectives.

6.2 Inclusion Criteria

Participants must meet all of the following criteria to be included in the study:

1. Male or female, ≥18 and ≤65 years of age, with BMI >18.5 and <32.0 kg/m^2.
2. Healthy as defined by:
   a. The absence of clinically significant illness and surgery within 4 weeks prior to study drug administration.
   b. The absence of clinically significant history of neurological, endocrine, cardiovascular, respiratory, hematological, immunological, psychiatric, gastrointestinal, renal, hepatic, and metabolic disease. Fully resolved BCC and SCC are acceptable.
3. Females of non-childbearing potential must be:
   a. post-menopausal (spontaneous amenorrhea for at least 12 months prior to dosing) with confirmation by documented FSH levels ≥40 mIU/mL;

   or

   b. surgically sterile (bilateral oophorectomy or hysterectomy) at least 3 months prior to dosing.
4. Sexually active females of childbearing potential and non-sterile males must be willing to use an acceptable contraceptive method throughout the study as detailed in section 8.1.
5. Male participants must be willing not to donate sperm for 120 days and female participants must be willing not to donate eggs for 120 days after dosing.

6. Willing to abstain from soft or hard drugs (except marijuana) from screening and throughout the study, and from alcohol-based products from 24 hours prior to screening and admission until discharge and from 24 hours prior to each follow-up visit, and from cigarettes or alternative nicotine products during confinement.
7. Non-tattooed, clear injection site (ie, absence of dermatologic conditions, such as scarring or rash, that may impact the ability to assess injection site reactions) suitable for IV or IM injection and monitoring in the opinion of the Investigator.
8. Able to understand the study procedures and provide signed informed consent to participate in the study.

6.3 Exclusion Criteria

Participants to whom any of the following applies will be excluded from the study:

1. Any clinically significant abnormal finding at physical examination.
2. Clinically significant abnormal laboratory test results at screening or Day -1, or any positive serology test results for HBsAg, HCV antibody, or HIV antigen and antibody at screening. A single repeat for safety laboratory assessment to confirm initial result and trending is allowed per Investigator's discretion.
3. Positive pregnancy test or lactating female participant.
4. Positive urine drug screen (excluding marijuana), or alcohol breath test. Per Investigator's discretion, a single repeat for drug abuse urine test in the event of a false positive is allowed.
5. History of significant allergic reactions (e.g., anaphylactic reaction, hypersensitivity, angioedema) to any drug.
6. Clinically significant ECG abnormalities or vital signs abnormalities (systolic BP lower than 90 or over 140 mmHg, diastolic BP lower than 40 or over 90 mmHg, HR less than 40 or over 100 bpm, or RR less than 10 or over 22 bpm) at screening, or Day -1.
7. Any of the following laboratory parameters above the ULN values at screening, or Day -1: AST, ALT, direct bilirubin, indirect bilirubin, and total bilirubin. Only abnormal values up to 1.5x ULN may be repeated once for confirmation to below ULN. Isolated elevation in unconjugated bilirubin (e.g., suspected Gilbert's syndrome) is not exclusionary.
8. History of drug abuse within 1 year prior to screening.
9. History of alcohol abuse within 1 year prior to screening or regular use of alcohol within 6 months prior to screening that exceeds 14 units for women and 21 units for men of alcohol per week (1 unit = 200 mL of beer 5%, 83 mL of wine 12%, or 25 mL of distilled alcohol 40%).
10. Participants who smoke more than 10 cigarettes per day or the equivalent per week.
11. History of rare hereditary sucrose intolerance (e.g., genetic sucrose-isomaltase deficiency (GSID).

12. History of a known or suspected respiratory system disorder including, but not limited to, cystic fibrosis, interstitial lung disease, reactive airway disease, emphysema, chronic bronchitis, pulmonary hypertension, COPD, or asthma (participants with childhood asthma can be included in the study).
13. Diagnosis or suspected diagnosis of immunodeficiency or autoimmune diseases, or undergoing immunosuppressive therapy such as anticancer chemotherapy or radiotherapy before the study, or has received systemic corticosteroid treatment (topical corticosteroids are acceptable) within the past 120 days before dosing.
14. Poor peripheral venous access for Cohorts 1a, 2a, and 3a.
15. Fever (≥ 38.0°C) within 14 days before study drug administration.
16. Positive COVID-19 test at admission to the CRU.
17. Participants who had close contact (without PPE) as defined by the CDC in the past 14 days prior to screening, with someone diagnosed with SARS-CoV-2 infection or COVID-19 within 10 days of the close contact. Participants may be rescreened after 14 days provided that they remain asymptomatic.
18. Participants who have been infected by COVID-19, within 120 days prior to PDI204 administration.
19. Vaccination with COVID-19 vaccine within 120 days prior to administration of PDI204 until EOS or ET.
20. Vaccination (other than COVID-19 vaccine) within 30 days prior to administration of PDI204 until 30 days post-dose.
21. Known or suspected intolerance or hypersensitivity to any biologic medication or known allergies or clinically significant reactions to human proteins, mAbs or antibody fragments, or to any components of the formulation of PDI204 and its excipients used in this study.
22. History of bleeding disorders or clotting disorders (e.g., hemophilia, thrombocytopenia) or those with a history of easy bruising or bleeding who may be at a higher risk for hematoma at the injection site.
23. Has known active infection with influenza or other non-SARS-CoV-2 respiratory pathogen, confirmed by a diagnostic test.
24. Previous infusion-related reaction.
25. Use of systemic immunosuppressive or immunomodulatory therapies during the study period.
26. Use of medications within the timeframes specified in section 8.2.

27. Participation in a clinical research study involving the administration of an investigational or marketed drug (including mAbs) or device within 30 days (or 5 half-lives since last receipt of an investigational drug, whichever is longer) prior to the first dosing, administration of a biological product in the context of a clinical research study within 90 days prior to the first dosing, or concomitant participation in an investigational study involving no drug or device administration.
28. Donation of serum within 7 days prior to dosing or donation or loss of 500 mL or more of whole blood within 30 days prior to screening.
29. Any reason which, in the opinion of the Investigator, would prevent the subject from participating in the study.

6.4 Participant Withdrawal and Replacement

Participants will be advised that they are free to withdraw from the study at any time. Over the course of the study, the Sponsor and the Investigator or designee may withdraw any participant from the study for one of the reasons described below; participant withdrawal will be done in accordance with the clinical site's standard operation procedure (SOP):

- Safety reason;
- Non-compliance with protocol requirements;
- Significant protocol deviation;
- Positive pregnancy test, drug screen, or alcohol breath test.

Participants who withdraw prior to dosing may be replaced automatically by the clinical site. Participants who withdraw or are withdrawn from the study after dosing, for reasons other than safety and tolerability, may be replaced at the discretion of the Sponsor and the Investigator or designee. In such case, the total number of participants dosed will remain within a maximum of 10 participants per cohort.

Participants who withdraw or are withdrawn will be asked to remain at the clinic until the Investigator or a delegate agrees that the participant is fine and can be discharged. A PK blood draw may be collected at the time of withdrawal if deemed required by the Investigator. Early termination (ET) procedures will be performed at the time of withdrawal from the study or as soon as possible thereafter.

7 STUDY DRUGS

7.1 Drug Supplies and Accountability

It is the responsibility of the Sponsor to ensure that the study drugs provided for this study are manufactured under Good Manufacturing Practice (GMP) and are suitable for human use. The Sponsor is responsible to ship a sufficient amount of dosage units to allow the clinical site to maintain an appropriate sampling for the study.

The study drugs will be stored at the clinical site pharmacy as per applicable requirements in a locked, temperature-controlled medication room with restricted access. Container(s) will be labeled according to applicable regulations.

Individual doses for each participant will be dispensed at the clinical site pharmacy, as per appropriate SOP, and in accordance with Pharmacy Manual, and according to the randomization scheme, in appropriate envelopes/containers that will be identified with at least the protocol number and the participant randomization number.

All study drugs received at the site will be inventoried and accounted for throughout the study and the result recorded in the drug accountability record according to the clinical site appropriate SOP.

7.2 Identification of Study Drugs

Active IP: PDI204 100 mg/mL vial for IM injection or IV infusion diluted with 0.9% saline (manufactured by Sypharma – Australia for The University of Melbourne)

Placebo: 0.9% saline vial identical to the active PDI204 (manufactured by Sypharma – Australia for The University of Melbourne)

A separate Pharmacy Manual will detail the storage, dispensing, and treatment administration of the active IP and placebo to be followed for this study.

7.3 Randomization and Blinding

For each cohort, participants eligible for participation will be randomized (on Day -1 or Day 1) to receive either the active or placebo in a 3:1 ratio, for a total of 6 participants receiving PDI204 and 2 participants receiving the placebo in each dose level. For each dose level, a staggered dosing schedule will be used and will include 2 sentinel participants randomized in a 1:1 ratio (1 active and 1 placebo) dosed initially, and the remaining 6 participants (5 actives and 1 placebo) dosed at least 24 hours later in Cohorts 1a, 2a, and 3a (IV administrations); and at least 48 hours later in Cohort 2b (IM administration). One randomization scheme will be produced for each cohort separately.

The study will be double-blinded. The participants and the clinical personnel involved in the collection, monitoring, revision, or evaluation of AEs, or personnel who could have an impact on the outcome of the study will be blinded with respect to the participant's treatment assignment (PDI204 or placebo). Blinding will be maintained until at least the clinical phase of the study is completed, i.e., when reporting and evaluation of all AEs have been completed for all cohorts.

Designated unblinded pharmacy personnel at the clinical site not directly involved with the clinical aspects of the study will prepare, store, and dispense the study drug in a blinded manner, according to the randomization scheme. The active and placebo will have the same visual appearance in order to avoid compromising the study blinding.

If discernible differences between the active IP and placebo are identified, unblinded personnels may be assigned for drug administration. To maintain the study blind, procedures such as participant blindfolding during administration may be implemented when necessary.

Blinded PK results will be available during the course of the study. The results will be reported without revealing the participant's treatment assignment.

An envelope for each randomization number containing a participant's treatment assignment will be stored in a secure storage area. In the event that unblinding is required for a given participant, the Investigator may break the blind for a participant and should contact the Sponsor prior to unblinding the participant's treatment assignment, whenever possible. However, in the event of a medical emergency for an individual participant for whom knowledge of the study treatment is critical, the Investigator may break the blind immediately for that participant and a notification will be made to the Sponsor within 24 hours. The date and reason for unblinding will be recorded in the study source documents.

7.4 Study Drug Administration

Participants in each cohort will be administered their assigned treatment according to the randomization scheme and the planned dose levels described in Table 2.

7.4.1 Study Drug Administration – IV (Cohorts 1a, 2a, and 3a)

Dosing for Cohorts 1a, 2a, and 3a will be conducted at the clinical site in the morning of Day 1 via peripheral IV infusion with 0.9% saline to the participant's forearm veins in a supine position. Participants in each cohort will be administered their assigned treatment at the planned dose levels described in Table 2. Time of dosing will be set equal to the time when the IV infusion is completed.

The IV pumps should be programmed immediately prior to each dose and require a second staff member to verify the correct settings have been programmed compared to the Project Specific Procedure (PSP).

The infusion will be performed over a period of 30 (± 5) minutes. The begin infusion time and end infusion times of dose administration will be recorded, and all post dose study procedures will be scheduled based on the end of infusion. Any interruptions in dose administrations (stop/restarts, occlusion, pump failure, etc.) should be recorded in the case report form (CRF).

Refer to the IB and the Pharmacy Manual for the detailed preparation and administration procedures.

7.4.2 Study Drug Administration – IM (Cohort 2b)

Dosing for Cohort 2b will be conducted at the clinical site in the morning of Day 1. Each participant will receive two IM injections of 1.5 mL each, administered into the ventro-gluteal region on both sides in side-lying (lateral recumbent) or prone positions. Participants in Cohort 2b will be administered their assigned treatment at the planned dose level described in Table 2. Time of dosing will be set equal to the time when IM injection is completed. Refer to the Pharmacy Manual for the detailed preparation and administration procedures.

8 STUDY RESTRICTIONS

8.1 Contraception

Female participants who are of non-childbearing potential will not be required to use contraception. Male and female participants who practice abstinence from sexual intercourse or are continuously not heterosexually active as a usual and preferred lifestyle will not be required to use contraception, as well. However, women of childbearing potential must still undergo pregnancy testing as per protocol.

Female participants of childbearing potential who are sexually active with a non-sterile male partner (sterile male partners are defined as men vasectomized for at least 3 months prior to dosing) must be willing to use one of the following acceptable double barrier contraceptive methods for at least 120 days after the drug administration:

- Simultaneous use of hormonal contraceptive (e.g., oral, depot injection, implant, vaginal ring, intrauterine device) or non-hormonal intrauterine device for at least 4 weeks prior to dosing (must agree to use the same contraceptive throughout the study) or bilateral tubal occlusion combined with condom for the male partner. Hormonal contraceptives should begin at least 1 month prior to screening to ensure contraceptive is in full effect.

Male participants who are not vasectomized for at least 3 months prior to dosing, and who are sexually active with a female partner of childbearing potential must be willing to use one of the following acceptable contraceptive methods from the admission and for 120 days after the drug administration:

- Simultaneous use of condom combined with hormonal contraceptive (e.g., oral, depot injection, implant, vaginal ring, intrauterine device) or non-hormonal intrauterine device used for at least 4 weeks prior to sexual intercourse for the female partner.

Male participants (including men who have had a vasectomy) with a pregnant partner must agree to use a condom from the admission and for 120 days after the drug administration. Male participants with potentially postmenopausal partners who are under the age of 55 years must use condoms unless their partner's postmenopausal status has been confirmed by FSH level.

Male participants must also be willing not to donate sperm for 120 days after the drug administration.

8.2 Concomitant Medications

Participants will be required to avoid using medications for the timeframes specified below and throughout the study:

- Any drug known to induce or inhibit hepatic drug metabolism for 30 days prior to dosing;
- Prescription medications 48 hours prior to dosing through CRU discharge on Day 2. Stable medications (e.g. statin) may be permitted on a case-by-case basis;
- Any systemic immunosuppressants or immune-modifying drugs, from screening through EOS or ET;

- COVID-19 vaccine (including investigational or licensed vaccines), for 120 days prior to dosing until EOS or ET;
- Vaccines other than COVID-19 vaccine, within 30 days prior to administration of PDI204 until 30 days post-dose;
- OTC medications and natural health products (including herbal remedies such as St. John's wort, homeopathic and traditional medicines, probiotics, food supplements such as minerals, amino acids, essential fatty acids, and protein supplements used in sports) within 24 hours before each PK sampling (with the exception of contraception, occasional use of acetaminophen up to 2 g daily, and standard dose of multivitamins).

No concomitant medications will be allowed during the study, with the exception of hormonal contraceptives, medications required for the medical management of an AE, and medications exempted by the Investigator on a case-by-case basis that are judged unlikely to affect the PK profile of the study drug or participant safety (e.g., topical drug products without significant systemic absorption).

If vaccination is required for any reason, it must first be discussed with and exempted by the Investigator on a case-by-case basis to ensure that it does not compromise the PK profile of the study drug or the participant safety.

Medications taken by participants before dosing will be documented as prior medications and medications taken by participants after dosing through the last study day will be documented as concomitant medications. Any prior or concomitant medication use, other than the allowed medications stated above, will be reviewed and evaluated on a case-by-case basis by the Investigator to determine if they affect a participant's eligibility or continued participation in the study, or for potential impact on the study results.

8.3 Drugs, Nicotine and Alcohol

Participants will be required to abstain from:

- Soft or hard drugs (except marijuana) from screening and throughout the study;
- Cigarettes or alternative nicotine products during confinement at the study site; No more than equivalent of 10 cigarettes per day will be allowed after discharge until EOS or ET;
- Alcohol-based products from 24 hours prior to screening and admission until discharge and from 24 hours prior to each follow-up visit.

8.4 Diet

Participants will be required to abstain from:

- Energy drinks from 48 hours prior to dosing until discharge;
- Food containing poppy seeds from 24 hours prior to screening and admission.

8.5 Posture and Physical Activity

Participants will be required to remain seated or semi-reclined and avoid lying down or sleeping for 30 minutes post-dose/post end of infusion (EOI). Participants will be allowed to engage in normal activity at all other times.

Failure of participants to comply with these requirements does not constitute a deviation from the protocol if it is medically necessary, required for procedures, or to go to the bathroom. When appropriate, participants will be accompanied by a staff member while walking. Participants will be required to refrain from strenuous exercise at least 3 days before admission and 3 days before each follow-up visit.

9 STUDY PROCEDURES

Participants must provide written informed consent prior to initiation of any study procedures.

Unless otherwise specified, study procedures will be conducted in accordance with clinical site SOPs. From screening through the EOS, participants will undergo study procedures at the times specified in Table 1, and as described in sections below.

Every effort will be made to schedule and perform the procedures as close to the nominal time as possible, giving considerations to appropriate posture conditions, practical restrictions, and other procedures to be performed at the same time point.

Unless otherwise specified or for participant safety, when the timing of multiple procedures coincides, the following priority order should be adhered to, whenever possible: physical examination, vital signs, ECGs, serum and saliva samples for PK and immunogenicity analysis, and clinical laboratory samples.

9.1 Safety and Tolerability Assessments

Participants will be monitored throughout the study by the clinical staff for AEs. Adequate medical surveillance will be assured during the confinement period and the Investigator or designee will be available on call at all times. If necessary, the Investigator or designee at the clinical site or a healthcare professional in a nearby hospital will administer treatment for any AE(s). A crash cart or emergency bag containing the necessary rescue material and appropriate medications will be available in the clinic to allow rapid intervention in case of emergency.

Safety parameters will be assessed by the Investigator or designee, using the clinical site acceptance ranges as suggested guidelines in making the medical assessment.

For eligibility purposes, abnormal vital signs measurements or clinical laboratory test results may be repeated once if an abnormal result is observed at the initial reading. Moreover, abnormalities found in the ECG may need to be confirmed by repeated measurements. In the event that the participation of a participant in the study is delayed and some screening procedures had been performed outside of the prescribed screening window, outdated screening procedures can be repeated.

Safety assessments scheduled during the study will be repeated according to the clinical site SOPs or upon request from the Investigator or designee. Any abnormal repeated measurement

will be evaluated and repeated, if judged necessary. Further action may be taken upon the Investigator or designee's request.

9.1.1 Physical Examination

Physical examinations will be performed at the times specified in Table 1.

A complete physical examination will include assessments of the following: head, eyes, ears, nose, throat (HEENT), neck, chest, lungs, abdomen, musculoskeletal, dermatological, cardiovascular/peripheral vascular, and general neurological examination.

A brief physical examination will include assessments of the following: HEENT, chest, lungs, abdomen, dermatological, cardiovascular/peripheral vascular, and areas of note elicited from the participant.

9.1.2 Body Measurements

Body measurements will be performed at the times specified in Table 1, and will include body weight, height measurement, and BMI calculation.

9.1.3 Infusion/Injection Site Reaction Assessment

Local reactions at the infusion site (Cohorts 1a, 2a, and 3a) and at injection site (Cohort 2b) will be evaluated by a trained observer who will be blinded to treatment assignment at the times specified in Table 1. The extent of local reaction at the infusion and injection site will be graded using the scores described in Table 3. A global severity rating for injection site reactions will be included in the assessment of AEs.

Table 3: Infusion/ Injection Site Reaction Scores

None	0	No reaction
Mild	1	Easily tolerated erythema and/or light bruising and/or mild pain
Moderate	2	Distributing erythema with swelling and/or distributing bruising and/or distributing pain
Severe	3	Almost intolerable symptoms, or clinically definite skin necrosis, characterized by any of the following: oozing, weeping, skin breakdown, ulceration, scar formation

In addition, bleeding or fluid loss at the infusion site (Cohorts 1a, 2a, and 3a) and at injection site (Cohort 2b) will be documented. The diameter of any erythema or swelling will be measured (in mm) in two dimensions. Reactions <10 mm will not be listed as an AE unless it persists for more than 24 hours.

9.1.4 Vital Signs

Blood pressure, heart rate, respiratory rate, and oral temperature will be measured after the participants have been resting for at least 3 minutes in a supine position at the times specified in Table 1.

9.1.5 12-lead ECG

Single 12-lead ECG will be recorded after the participants have been resting for at least 3 minutes in a supine position at the times specified in Table 1.

9.1.6 Laboratory Assessments

Blood and urine samples for clinical laboratory assessments will be collected under fasting conditions according to the clinical site SOPs at the times specified in Table 1. The clinical laboratory assessments to be performed are listed in Table 4.

Table 4: Clinical Laboratory Assessments

<table>
<tr><th>Biochemistry</th><th>Hematology</th><th>Urinalysis</th></tr>
<tr><td rowspan="3">Albumin
Alkaline phosphatase
ALT
AST
Bicarbonate
Calcium
Chloride
Creatinine
Creatine kinase
eGFR (calculated using the MDRD equation)
GGT
Glucose
Lipase
Phosphorus
Potassium
Sodium
Total, direct and indirect bilirubin
Total protein
Blood Urea Nitrogen (BUN)

Lipid profile:
Total cholesterol
Triglycerides
HDL-C
LDL-C</td><td>Hematocrit
Hemoglobin
MCH
MCHC
MCV
Platelet count
RBC count
RBC distribution width
WBC count and differential:
• Basophils
• Eosinophils
• Lymphocytes
• Monocytes
• Neutrophils</td><td rowspan="3">Bilirubin
Blood (occult)
Color and appearance
Glucose
Ketones
Leukocyte esterase
Nitrite
pH
Protein
Specific gravity
Urobilinogen
Microscopic examination (in the event of abnormal findings, per safety lab standard procedures)</td></tr>
<tr><th>Coagulation</th></tr>
<tr><td>aPTT
PT
INR</td></tr>
<tr><th>Serology</th><th>Drug, and alcohol screens</th><th>Hormone panel - females only</th></tr>
<tr><td>HBsAg
HCV antibody
HIV antigen/antibody</td><td>Amphetamines/methamphetamines
Barbiturates
Benzodiazepines
Cocaine
MDMA
Methadone
Opiates
PCP

Alcohol breath test</td><td>FSH (post-menopausal females only)
Serum pregnancy test
Urine pregnancy test</td></tr>
</table>

Abbreviations: ALT = Alanine aminotransferase; aPTT = activated partial thromboplastin time; AST = Aspartate aminotransferase; BUN = blood urea nitrogen; eGFR = estimated glomerular filtration rate; FSH = follicle-stimulating hormone; GGT = gamma-glutamyl transferase; HBsAg = hepatitis B surface antigen; HCV = hepatitis C virus; HDL-C = high density lipoprotein cholesterol; HIV = human immunodeficiency virus; INR = international normalized ratio; LDL-C = low-density lipoprotein cholesterol; MCH = mean cell hemoglobin; MCHC = mean cell hemoglobin concentration; MCV = mean cell volume; MDMA = 3,4- methylenedioxymethamphetamine; MDRD = modification of diet in renal disease; PCP = phencyclidine; pH = potential of hydrogen; PT = prothrombin time; RBC = red blood cell; WBC = white blood cell.

9.2 Adverse Events

9.2.1 Definition of Adverse Event

An AE is defined as any untoward medical occurrence in a patient or clinical trial participant administered a pharmaceutical product and which does not necessarily have a causal relationship with the treatment. An AE can, therefore, be any unfavorable and unintended sign (including an abnormal laboratory finding, for example), symptom, or disease temporally associated with the use of a medicinal (investigational) product, whether or not considered related to the medicinal (investigational) product.

9.2.2 Recording of Adverse Events

AEs will be recorded and evaluated for their seriousness, severity, and relationship to the study drug. AEs will be collected and documented from the time of signing the informed consent form (ICF) until EOS or ET. AEs will be followed-up until complete resolution, or until the Investigator or designee judges safe to discontinue follow-up. The severity of AEs and relationship to the study drug will be classified according to CTCAE Version 5.0.

9.2.3 Assessment of Severity

The Investigator or designee will review each event (including infusion/injection site reactions) and assess its severity based on the National Cancer Institute's Common Terminology Criteria for Adverse Events (CTCAE), version 5.0. If a participant has an AE not listed in the CTCAE, the following grading system should be used to assess severity:

- Grade 1: Mild; asymptomatic or mild symptoms; clinical or diagnostic observations only; intervention not indicated.
- Grade 2: Moderate; minimal, local, or noninvasive intervention indicated; limiting age-appropriate instrumental activities of daily living.
- Grade 3: Severe or medically significant but not immediately life-threatening; hospitalization or prolongation of hospitalization indicated; disabling; limiting self-care activities of daily living.
- Grade 4: Life-threatening consequences; urgent intervention indicated.
- Grade 5: Death related to AE.

A mild, moderate, or severe AE may or may not be serious. These terms are used to describe the intensity of a specific event. Medical judgment should be used on a case-by-case basis. Seriousness, rather than severity assessment, determine the regulatory reporting obligations.

9.2.4 Assessment of Relationship to Study Drug

Each AE must be classified based on medical judgment and according to the following relationship categories: probable, possible, remote/unlikely, and unrelated. The definitions for the relationship categories are detailed in Table 5.

Table 5: Adverse Event Relationship Categories

Category	Description
**[Related] Probable (must have first three points)**	This category applies to AEs that are considered, with a high degree of certainty, to be related to the investigational product. An AE may be considered probable, if:<br>1. It follows a reasonable temporal sequence from the administration of the drug.<br>2. It cannot be reasonably explained by the known characteristics of the participant's clinical state, environmental or toxic factors or other modes of therapy administered to the participant.<br>3. It disappears or decreases on cessation or reduction in dose (there are important exceptions when an AE does not disappear upon discontinuation of the drug, yet drug relatedness clearly exists; e.g. (1) bone marrow depression, (2) tardive dyskinesias).<br>4. It follows a known pattern of response to the suspected drug.
**[Related] Possible (must have first two points)**	This category applies to AEs in which the connection with the investigational product administration appears unlikely but cannot be ruled out with certainty. An AE may be considered possible if, or when:<br>1. It follows a reasonable temporal sequence from the administration of the drug.<br>2. It may have been produced by the participant's clinical state, environmental or toxic factors, or other modes of therapy administered to the participant.<br>3. It follows a known pattern of response to the suspected drug.
**Remote/Unlikely (must have first two points)**	In general, this category is applicable to an AE that meets the following criteria:<br>1. It does not follow a reasonable temporal sequence from the administration of the investigational product.<br>2. It may readily have been produced by the participant's clinical state, environmental or toxic factors, or other modes of therapy administered to the participant.<br>3. It does not follow a known pattern of response to the suspected drug.<br>4. It does not reappear or worsen when the investigational product is re-administered.
**Unrelated**	This category is applicable to AEs that are judged to be clearly and incontrovertibly due only to extraneous causes (disease, environment, etc.), and do not meet the criteria for medication relationship listed under remote/unlikely, possible, or probable.

9.2.5 Definition of Serious Adverse Event

A SAE is defined as any untoward medical occurrence that, at any dose:

- Results in death;
- Is life-threatening;
- Requires inpatient hospitalization or prolongation of existing hospitalization;
- Results in persistent or significant disability/incapacity;
- Is a congenital anomaly/birth defect, or;
- Is otherwise considered to be an important medical event that may not be immediately life-threatening or result in death or hospitalization but may jeopardize the patient or may require intervention to prevent one of the other outcomes listed in the definition above. Medical and scientific judgement should be exercised in deciding whether an event should be considered as an Important Medical Event. Examples of such medical events include:
  - Allergic bronchospasm requiring intensive treatment in an emergency room or at home;
  - Blood dyscrasias or convulsions that do not result in hospitalization;
  - Development of drug dependency or drug abuse.

9.2.6 Reporting of Adverse Events

9.2.6.1 Serious Adverse Event Reporting to the Sponsor

Any SAE will be reported to the Sponsor (or representative) within 24 hours of learning of the event. The notification must be directed to:

SAE contact information

For medical emergencies, contact:

Medical Monitor: Jon Rankin, MB BS Grad. Dip. PH SCHP JD(Hons) LLM Grad. Dip. Legal Practice

Mobile: +61 499 222 117

E-mail: jon.rankin@syneoshealth.com

Information on SAEs will be recorded on the SAE Report Form. Blank copies are included in the study Investigator's file. It is not acceptable for the Investigator to send photocopies of the patient's medical records to the Sponsor company or its representative in lieu of completion of the appropriate AE eCRF page or SAE Report Form. However, there may be instances when copies of medical records for certain cases are requested by the Sponsor or its representative. In this instance, all participant identifiers will be blinded on the copies of the medical records before submission to the Sponsor or its representative.

The completed SAE Form and SAE cover sheet should be sent via fax or e-mail immediately upon completion to Syneos Health Safety and Pharmacovigilance. If the Investigator does not

have all information regarding an SAE, he/she will not wait to receive additional information before completing and sending the form.

Additional relevant information or clinical follow-up should be sent via fax or e-mail to Syneos Health Safety and Pharmacovigilance as soon as it becomes available. The Investigator should follow the participant with the event until resolution or stabilization of the condition. Follow-up reports (as many as required) should be completed and faxed/e-mailed following the same procedure above.

A final report is required once the condition is resolved or stabilized and no more information about the event is expected. The final report should be completed and faxed/e-mailed following the same procedure above.

The Investigator must keep a copy of all documentation related to the event in the clinical site files.

If the Investigator learns of any SAE, including death, at any other time after a participant completes the study, and he/she considers the event reasonably related to the study drug, the Investigator will promptly notify the Sponsor/Medical Monitor.

9.2.6.2 Serious Adverse Event Reporting to the IEC and Regulatory Agency(ies)

It is the responsibility of the clinical site to report suspected, unexpected, serious adverse reactions (SUSARs) to the IEC responsible for the study per their policies. Report of fatal or life-threatening SUSARs must be made as soon as possible, but no later than 7 calendar days after first knowledge of the event. Report of SUSARs that are neither fatal nor life-threatening must be made as soon as possible, but no later than 15 calendar days after first knowledge of the event.

The Sponsor (or representative) is responsible for notifying the regulatory agency(ies) of SUSARs observed during the study conduct per their regulations, as soon as possible but no later than 7 calendar days after becoming aware of the information when fatal or life-threatening, or 15 calendar days when neither fatal nor life-threatening. The Sponsor (or representative) is responsible to comply with any other applicable regulatory requirement(s) related to the reporting of SAE to other regulatory authority(ies).

9.2.7 Pregnancy

In the event a dosed female participant or the female partner of a dosed male participant (i.e., has received at least one dose of the investigational product) becomes pregnant during or shortly after participation in the study, this pregnancy will be reported to the Sponsor (or representative) within 24 hours of first knowledge of the event. Any participant who becomes pregnant during the study will be immediately withdrawn. Follow-up information regarding the course and outcome of the pregnancy will be documented (after obtaining the consent of the female partner) as per site’s SOP. If the outcome of the pregnancy meets the criteria of reportable event, reporting of the event to the IEC responsible for the study and/or to applicable regulatory agency(ies) will be performed as per site’s SOP.

9.2.8 Premature Termination of the Study

The study may be prematurely terminated by the Investigator following consultation with the Sponsor, by the Sponsor or by the regulatory authorities. Following a decision to discontinue the trial, the Investigator will promptly inform the active study participants and the IEC responsible for this trial, stating the reasons for discontinuation of the study. It is the responsibility of the Sponsor (or representative) to report the premature termination of the study to the regulatory authority(ies), when required by the applicable regulatory requirement(s).

9.3 Pharmacokinetic Assessments

9.3.1 Blood Sample Collection and Processing

Blood samples for PK analysis will be collected via an intravenous catheter or by direct venipuncture at time points indicated in Table 1. Applicable time windows for PK blood samples are defined in Table 6.

Table 6: Time Windows for PK Blood Samples

Day(s)	Time points	Time window
1	Pre-dose	Within 60 minutes
	30 minutes	±5 minutes
	4 hours	±15 minutes
	8, and 12 hours	±60 minutes
2	24 hours	±60 minutes
3, 5	48, and 96 hours	±180 minutes
7, 15	144, and 336 hours	±1 day
30, 60	696, and 1416 hours	±3 days
90	EOS	±7 days

Abbreviation: EOS= end of study.

Sample collections done outside the pre-defined time windows will not be considered as protocol deviations since actual post-dose sampling times will be used for PK, and statistical analyses.

No more than 500 mL of blood will be collected from the participant during their study participation. Additional tests or blood draws could be performed, if deemed required by the Investigator or study staff. Procedures for collection, processing, and shipping of blood samples for PK will be detailed in a separate document.

The SRC may recommend to modify the PK sample collection schedule based on emerging data, including shortening or lengthening the total collection interval by up to 24 hours, and may add up to two additional PK serum samples, if necessary.

9.4 Immunogenicity Assessments

9.4.1 9.4.1 Anti-drug antibodies (ADAs) Assessment

Serum will be collected according to Table 1 for analysis of immunogenic response, using ELISA assay, to determine presence/absence and titers of ADAs. Applicable time windows for ADAs blood samples are defined in Table 7.

Table 7: Time Windows for ADAs Blood Samples

Day(s)	Time points	Time window
1	Pre-dose	Within 60 minutes
30	696 hours	±3 days
90	EOS	±7 days

Abbreviation: EOS= end of study.

The presence of ADAs and the possible impact that their presence has on PK parameters or safety may be investigated as appropriate and as data permit.

9.4.2 SARS-CoV-2 Neutralizing antibodies (NAbs) Assessment

Serum and saliva samples will be collected to characterize NAbs against SARS-CoV-2, as appropriate, using a live virus neutralization assay, at timepoints described in Table 1. Applicable time windows for ADAs blood samples and Nab blood and saliva samples are defined in Table 8.

Table 8: Time Windows for Nabs Blood and Saliva Samples

Day(s)	Time points	Time window
1	Pre-dose	Within 60 minutes
2	24 hours	±60 minutes
3	48 hours	±180 minutes
7, 15	144, and 336 hours	±1 day
30	696 hours	±3 days
90	EOS	±7 days

Abbreviation: EOS= end of study.

9.5 Analytical Methods

Serum and saliva samples from participants randomized to PDI204 will be assayed even if the participants do not complete the study. Samples will be analyzed for PDI204 concentrations, ADAs, and NAbs using validated bioanalytical methods.

Analysis of ADA samples will be performed using a validated assay in a 3-tier format (screen, confirm, titer).

Details of the analytical methods will be provided in a separate document.

10 STATISTICAL ANALYSES

A complete description of the statistical analyses to be performed on safety and tolerability data, as well as PK data will be presented in a statistical analysis plan (SAP).

10.1 Analysis Populations

10.1.1 Safety Population

The safety population will include all participants who receive at least one dose of the study drug PDI204 or placebo). The safety population will be used for the summaries of all safety assessments.

10.1.2 Pharmacokinetic Population

The PK population will include all participants from the safety population for whom the PK profile can be adequately characterized.

10.1.3 Immunogenicity Population

The immunogenicity population will include all participants from the safety population for whom the immugenicity profile can be adequately characterized.

10.2 Safety and Tolerability Parameters

Safety and tolerability of PDI204 will be evaluated through the assessment of AEs (i.e., seriousness, severity [using the current CTCAE, version 5.0], relationship to the study drug, outcome, duration, and management), SAEs, vital signs, 12-lead ECGs, clinical laboratory tests, physical examinations, and injection site reaction assessment. AEs will be coded using the latest version of the MedDRA.

10.3 Safety and Tolerability Statistical Analyses

Demographic parameters will be summarized descriptively.

Safety and tolerability analysis will be performed for all participants in the safety population. No inferential statistical analysis of safety data is planned.

TEAEs will be tabulated by treatment. Changes from baseline values in vital signs, 12-lead ECGs, and clinical laboratory tests will be evaluated. Safety and tolerability data will be reported using descriptive statistics. The TEAE rates will be summarized by SOC and preferred term (PT).

10.4 Pharmacokinetic Parameters

Preliminary PK analyses will be performed after completion of each dose level, without revealing the participant numbers in order to preserve the blind.

The following PK parameters will be calculated for PDI204 serum concentrations:

PK parameters:

- AUC_{0-t}: Area under the concentration-time curve from time zero until the last observed concentration
- AUC_{0-inf}: Area under the concentration-time curve from time zero to infinity (extrapolated)
- Residual area: Percentage of AUC_{0-inf} due to extrapolation from the time of the last observed concentration to infinity, calculated as $[1 - (AUC_{0-t}/AUC_{0-inf})] \times 100$
- C_{max}: Maximal observed concentration
- T_{max}: Time when the maximal concentration is observed
- $T_{½ el}$: Terminal elimination half-life
- K_{el}: Terminal elimination rate constant
- Cl: Total clearance (for IV treatments)
- V_z: Apparent volume of distribution during the terminal phase (for IV treatments)
- Cl/F: Apparent clearance (for IM treatment)
- V_z/F: Apparent volume of distribution (for IM treatment)

Additional PK parameters may be calculated.

10.5 Pharmacokinetic Statistical Analyses

Individual and mean serum concentration versus time curves will be presented for both linear and semi-log scales. Descriptive statistics (arithmetic and geometric means, standard deviation [SD], coefficient of variation [CV%], minimum [Min], maximum [Max], and median) of the serum concentrations versus time will be presented as well for the PK parameters.

Summary statistics will be used to describe the PK profile of PDI204 for each dose level.

The power model approach will be performed on AUC_{0-t}, AUC_{0-inf}, and C_{max} data to assess the dose-proportionality. Statistical comparisons of dose-normalized AUC_{0-t}, AUC_{0-inf} and C_{max} will be performed for IV treatments vs IM treatment.

10.6 Immunogenicity Assessments

10.6.1 Anti-Drug Antibodies (ADAs) Analyses

The incidence and prevalence of ADAs will be summarized by dose and visit, using descriptive statistics. The impact of ADAs on the PK of PDI204 will be assessed by comparing PK parameters (e.g., AUC_{0-t}, AUC_{0-inf}, and C_{max}) between ADA-positive and ADA-negative participants. Additionally, potential effects of ADA development on safety and serum levels of PDI204 might be explored.

10.6.2 SARS-CoV-2 Neutralizing Antibodies (NAbs) Analyses

Descriptive statistics will be used to summarize SARS-CoV-2 NAbs titers over time in serum and saliva. GMTs and corresponding 95% confidence intervals will be calculated for each time point. Longitudinal analyses may be conducted to assess the durability of the immune response,

and statistical comparisons between dose groups may be performed using an appropriate model (e.g., mixed-effects model or ANOVA).

11 DATA COLLECTION

The source data will be collected on paper as per site SOPs. Source data should be attributable, legible, contemporaneous, original, accurate, and complete. Source documents will be maintained in order to maintain data integrity. The Investigator and/or the clinical staff have the responsibility of ensuring the accuracy, completeness, legibility, and timeliness of the source data.

Details on the data management process will be described in a data management plan (DMP).

12 REGULATORY CONSIDERATIONS AND QUALITY ASSURANCE

12.1 IEC Approval of Protocol and Other Study Documents

The Investigator agrees to provide the IEC with all appropriate documents, including a copy of the protocol/amendments, ICFs, advertising text (if any), IB, and any other written information provided to study participants. The trial will not begin until the Investigator have obtained the IEC favourable written approvals for the above-mentioned study documents. A properly executed written ICF shall be read, signed, and dated by each participant prior to entering the trial or prior to performing any study procedure. The original signed and dated ICF will be kept at the clinical site and a copy will be given to the participant.

In the event that the protocol is amended, the revised protocol must be approved by the IEC prior to its implementation, unless the changes involve only logistical or administrative aspects of the trial. If a revised ICF is introduced during the study, each participant's further consent must be obtained. The new version of the ICF must be approved by the IEC, prior to subsequently obtaining each participant's consent.

The Investigator and the Sponsor's representative must sign the protocol and its amendments (if any) before initiating the study.

It is the Sponsor's responsibility to submit the protocol and its amendments (if any), and the ICFs to regulatory authorities when necessary.

12.2 Compliance

This study will be conducted in compliance with the protocol, Good Clinical Practice (GCP), all applicable regulations, and any IEC requirements relative to clinical studies. The study will also be conducted in compliance with the recommendations laid down in the most recent version of the Declaration of Helsinki, with the exception that registration of such Phase 1 trials in a publicly accessible database is not mandatory.

12.3 Quality Assurance and Monitoring

The Sponsor or its designee will perform the quality assurance and quality control activities of this study; however, responsibility for the accuracy, completeness, security, and reliability of the

study data presented to the Sponsor lies with the Investigator generating the data. When applicable, quality assurance procedures will be performed according to the site SOPs.

The study will be monitored according to the site monitoring plan and SOP to ensure that it is conducted and documented properly according to the protocol, GCP, and all applicable regulatory requirements. The Sponsor is entitled to access information about the status of the study and to review the original documents of the study.

The Sponsor may arrange audits as part of the implementation of quality assurance to ensure that the study is being conducted in compliance with the protocol, SOPs, GCP, and all applicable regulatory requirements. In such case, audits will be independent of and separate from the routine monitoring and quality control functions.

12.4 Confidentiality and Retention of Study Records

This document contains trade secrets and commercial information that is confidential and may not be disclosed to third parties. Persons to whom this study protocol is disclosed must be informed that all the information herein is confidential and may not be further divulged. These restrictions will apply as well to all future communications if deemed privileged or confidential. Publication of the study results may only be allowed with written permission from the Sponsor.

All information on a participant obtained during the conduct of the study will be kept confidential. Participants will be identified by an anonymized identifier on all samples and study records provided to the Sponsor or designee. The collection of race and ethnicity data from participants may be performed during the study in order to meet the study objectives and better interpret safety and PK data. In compliance with International Council for Harmonisation of Technical Requirements for Pharmaceuticals for Human Use (ICH) GCP, the Sponsor's authorized representatives, monitor(s), auditor(s), IEC, and regulatory authority(ies) will be granted direct access to the participant's original trial-related records for verification of clinical trial procedures and/or data, without violating the confidentiality of the participant, to the extent permitted by the applicable laws and regulations. Consent from the participant for disclosure of such information will be obtained in writing in the ICF. In addition, should a participant require medical care or hospitalization during the course of the study, the clinical site may contact the treating physician with the participant's consent, except that consent may not be requested if there is an emergency situation. If the results of the study are published, the participant's identity will remain confidential.

The clinical sites will maintain adequate study records according to applicable regulatory requirements. The Sponsor will be notified prior to the destruction of study records.

13 REFERENCES

1 PDI204. Investigator’s Brochure. The University of Melbourne. Version 1.0 [08 MAY 2025].

2 Center for Drug Evaluation and Research (CDER), FDA. Guidance for Industry: Estimating the Maximum Safe Starting Dose in Initial Clinical Trials for Therapeutics in Adult Healthy Volunteers. July 2005.

3 Committee for Medicinal Products for Human Use (CHMP), EMA. Guideline on strategies to identify and mitigate risks for first-in-human and early clinical trials with investigational medicinal products. EMEA/CHMP/SWP/28367/07 Rev. 1. 20 July 2017.
