# Supplementary material for: Randomised controlled phase I trial of a SARS‐CoV‐2 monoclonal antibody
Source: Clin Transl Immunology. 2026 Nov 4;15(11):e70126. doi: 10.1002/cti2.70126 (PMC13735506; doi:10.1002/cti2.70126)
Supplement: Supplementary file 2 [file CTI2-15-e70126-s003.pdf]

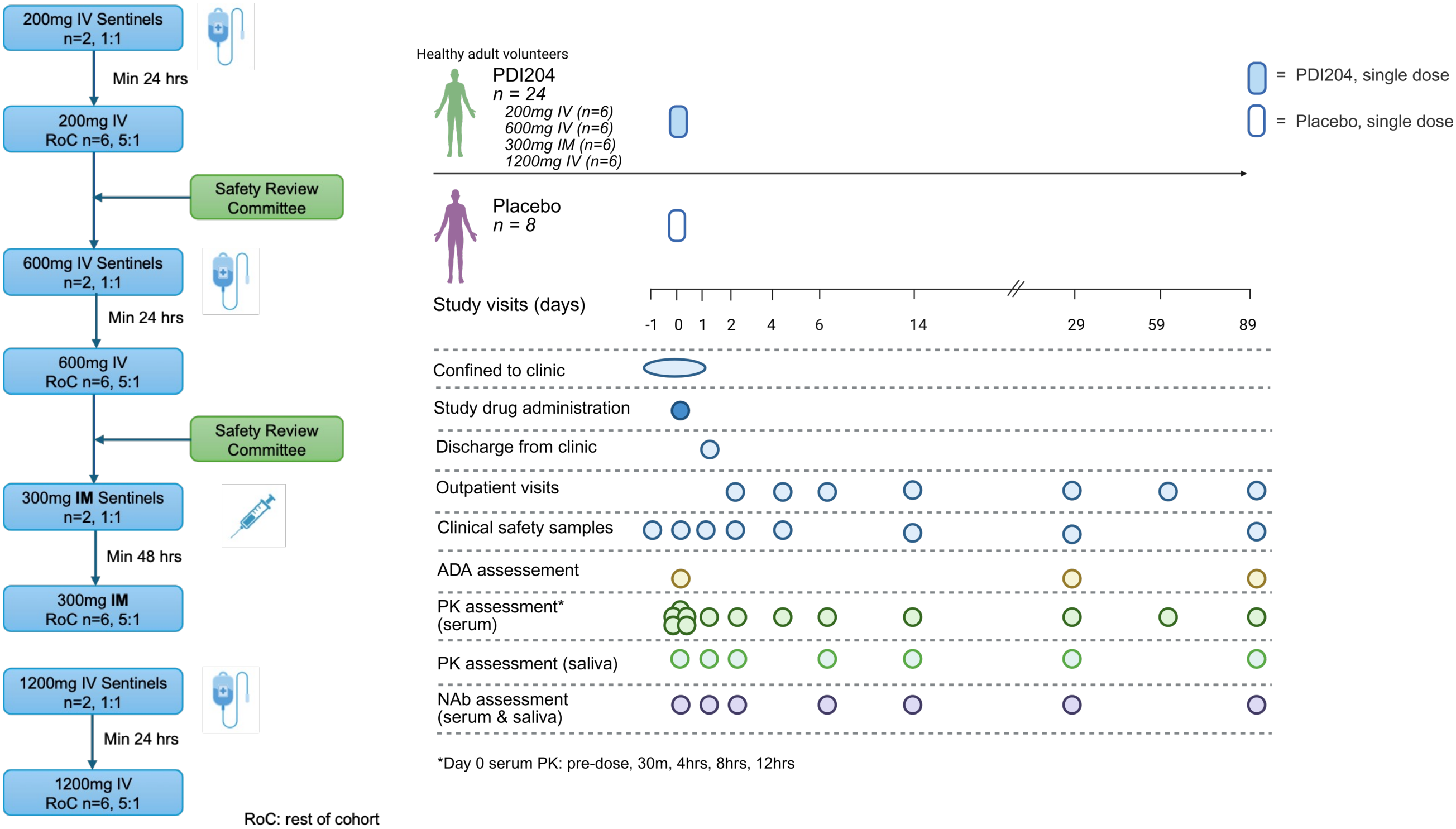

Supplementary Figure 1: Study Design and schedule of events

PDI204 Pharmacokinetics

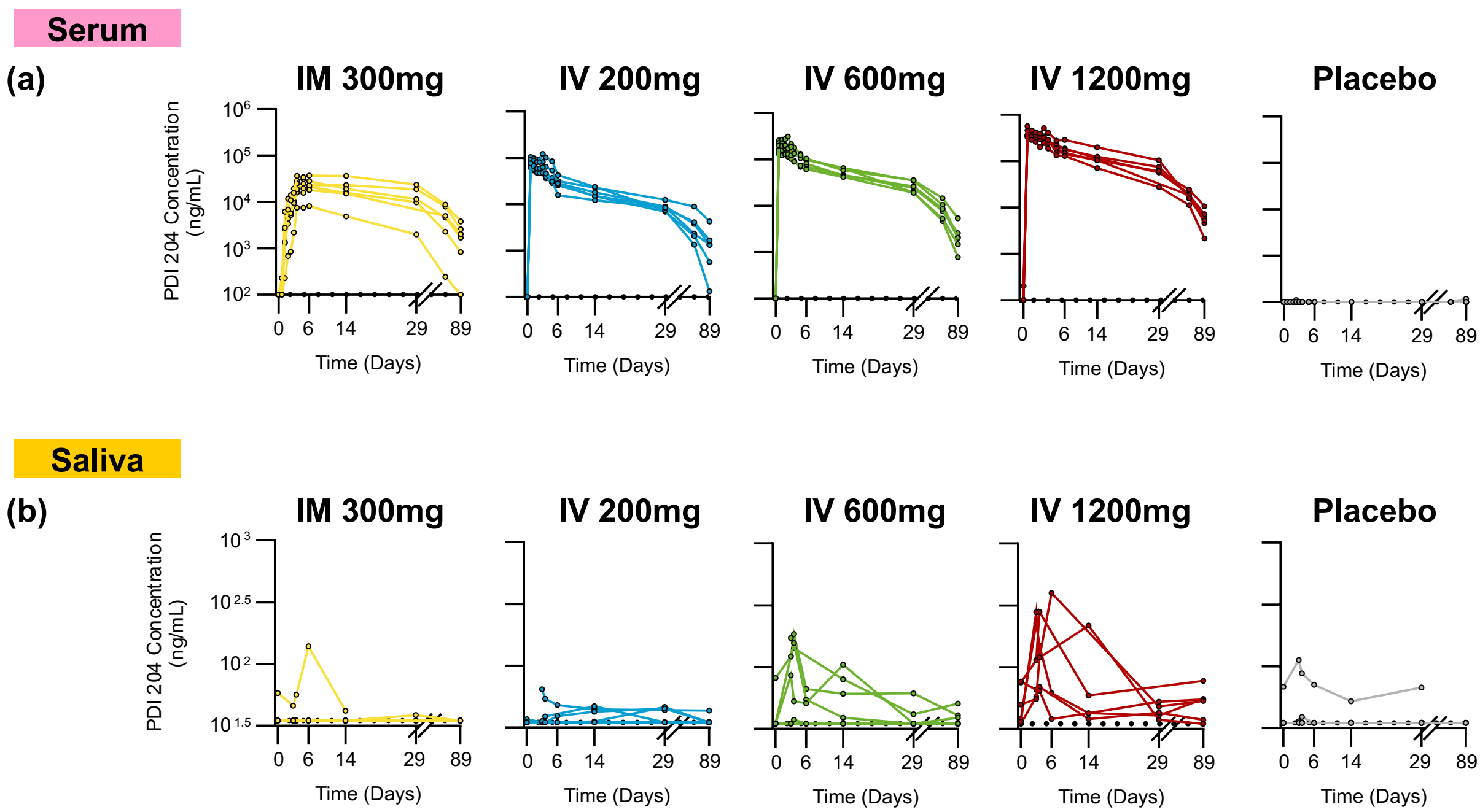

Supplementary Figure 2: Pharmacokinetics of PDI204 in serum and saliva. Concentrations of PDI204 measured for each study participant's **(a)** serum and **(b)** saliva at respective timepoints (n=6 for each IV and IM group, n=8 for placebo group). PDI204 concentration was measured via capture ELISA for serum and a bead-based multiplex assay for saliva. Dotted lines indicate the limit of detection. Assays were performed in duplicate.

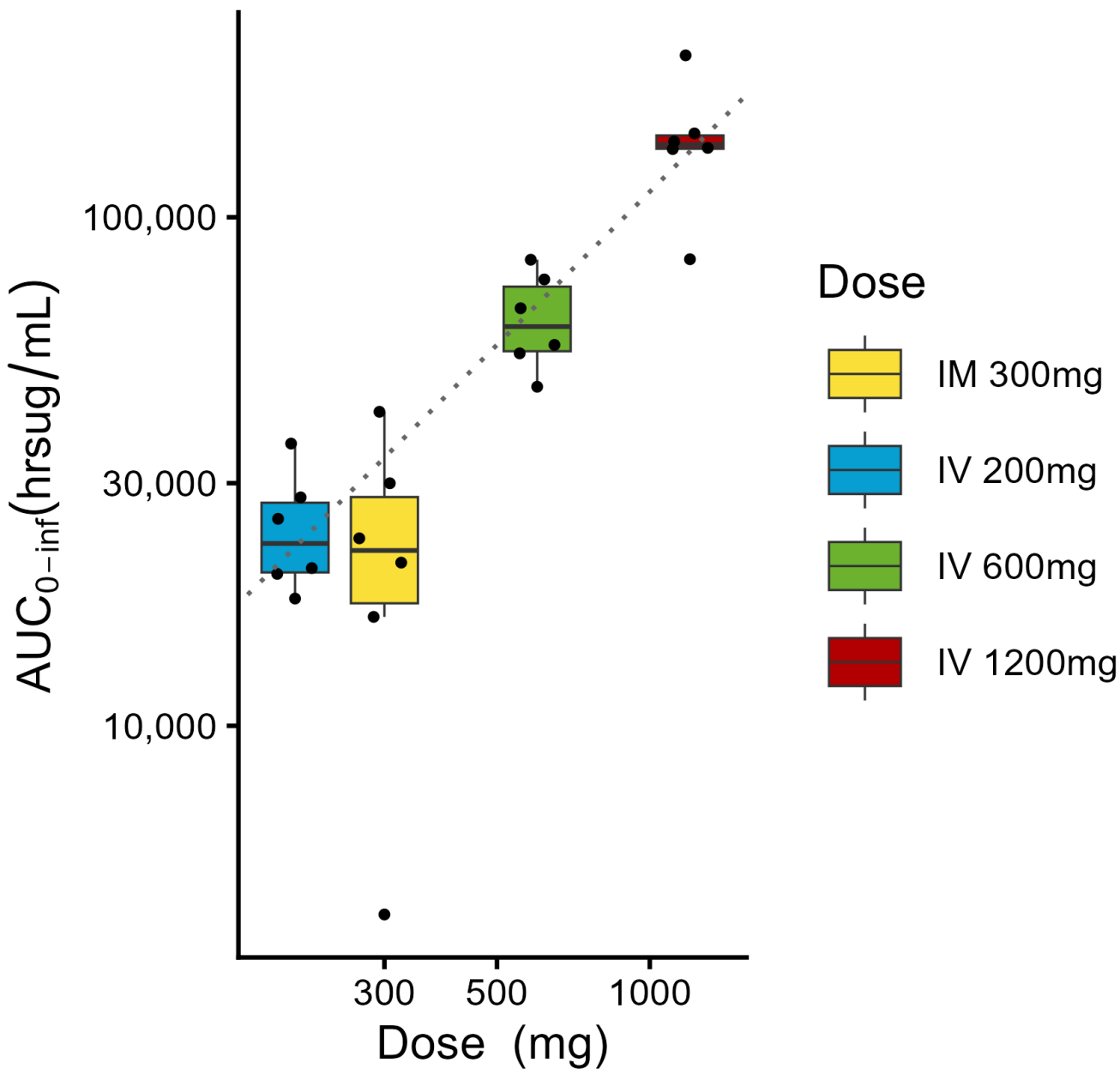

Supplementary Figure 3: Drug exposure (AUC$_{0\text{-}inf}$ (hrs µg/ml)) against dose of PDI204 administered.

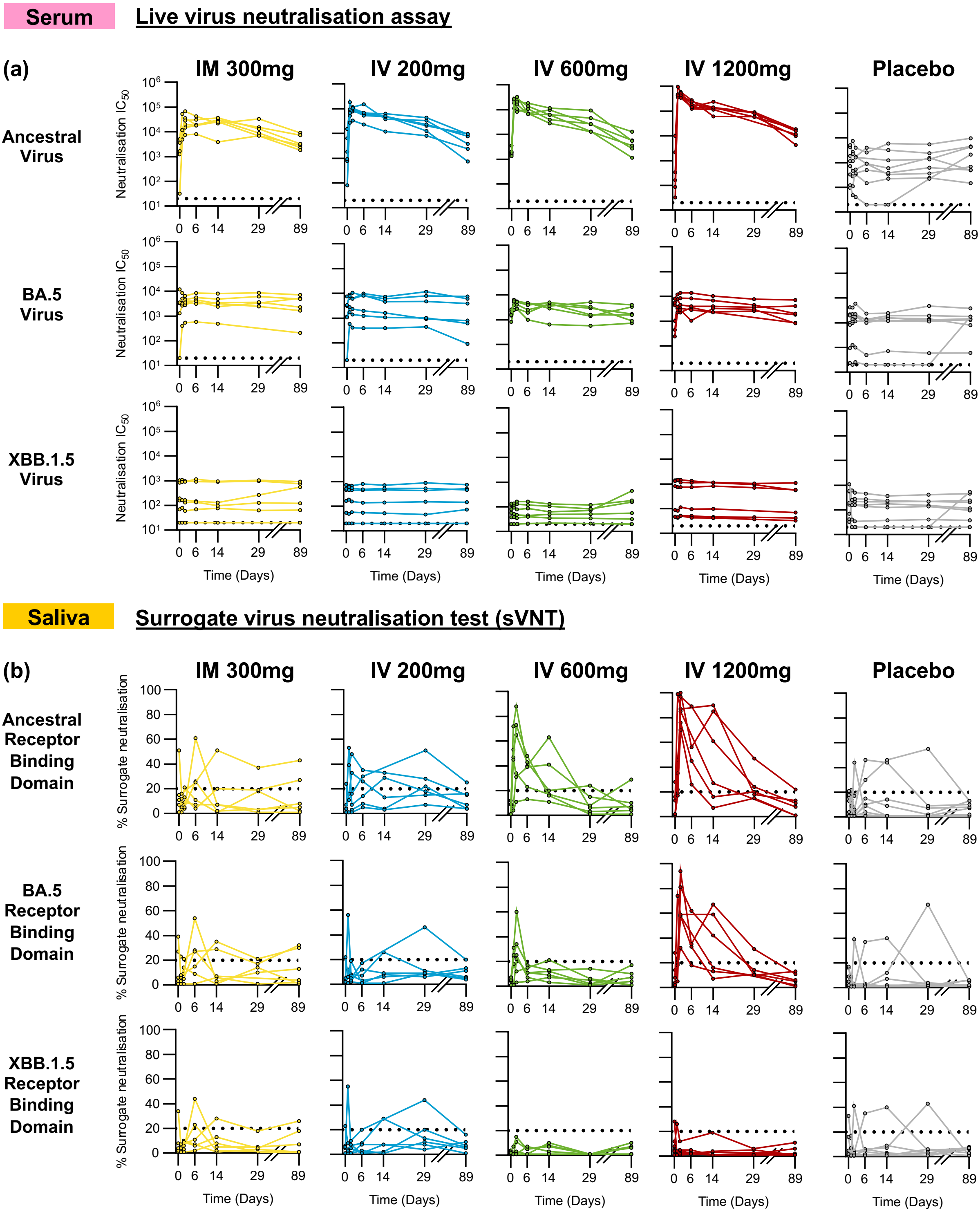

Supplementary Figure 4: Neutralizing activity in serum and saliva. Individual participant **(a)** serum and **(b)** saliva neutralizing responses against ancestral, BA.5 and XBB.1.5 across timepoints using live virus and surrogate neutralisation assays respectively (n=6 for each IV and IM group, n=8 for placebo group). Dotted lines indicate the cut-off values of the respective assays.

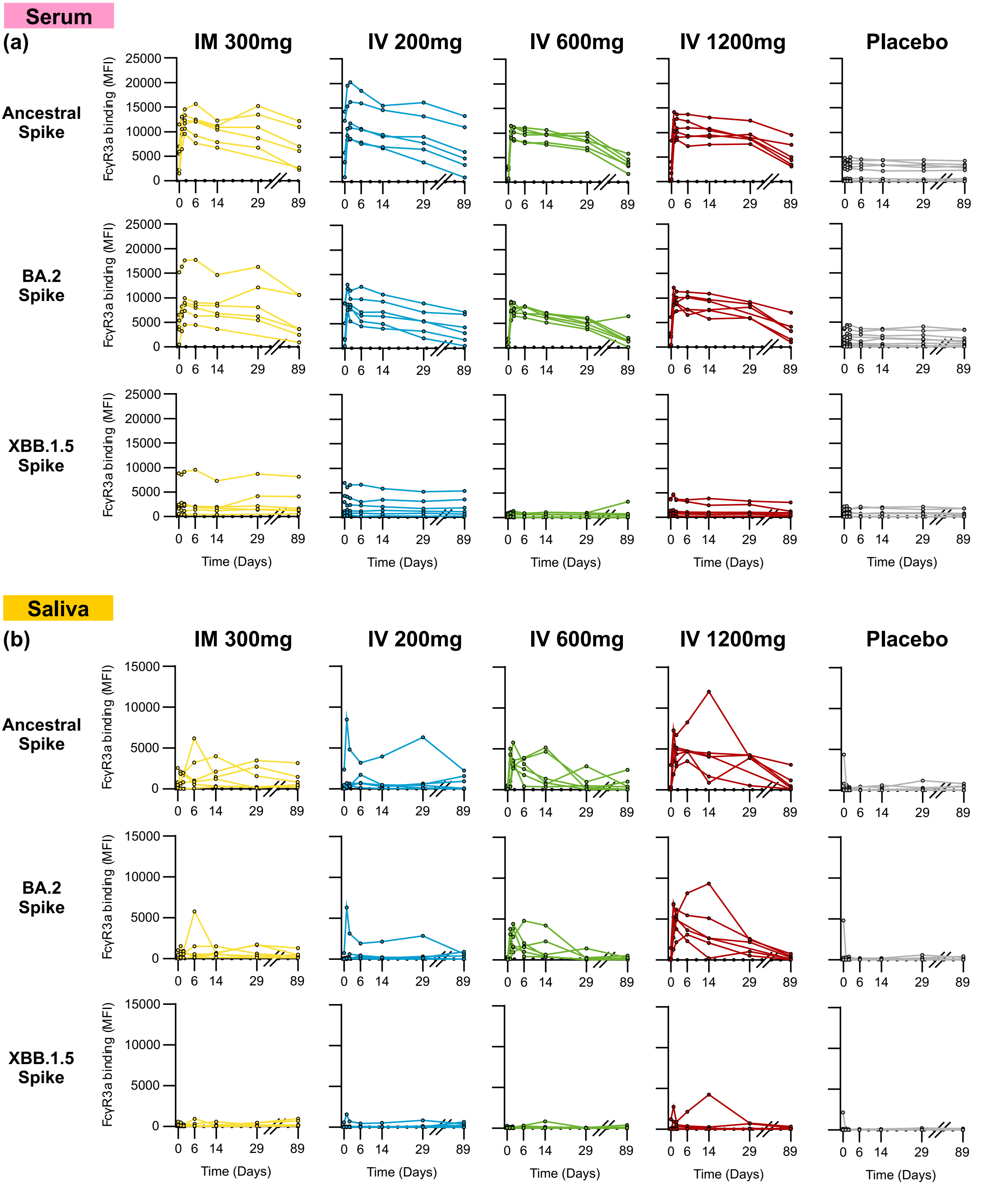

Supplementary Figure 5: FcγR3a-binding responses in serum and saliva. Binding of ancestral, BA.2 and XBB.1.5 spike-specific antibodies in each study participant's (a) serum and (b) saliva to FcγR3a across timepoints, measured via a bead-based multiplex assay (n=6 for each IV and IM group, n=8 for placebo group). Dotted lines indicate the background responses of pre-pandemic samples. Assays were performed in duplicate.

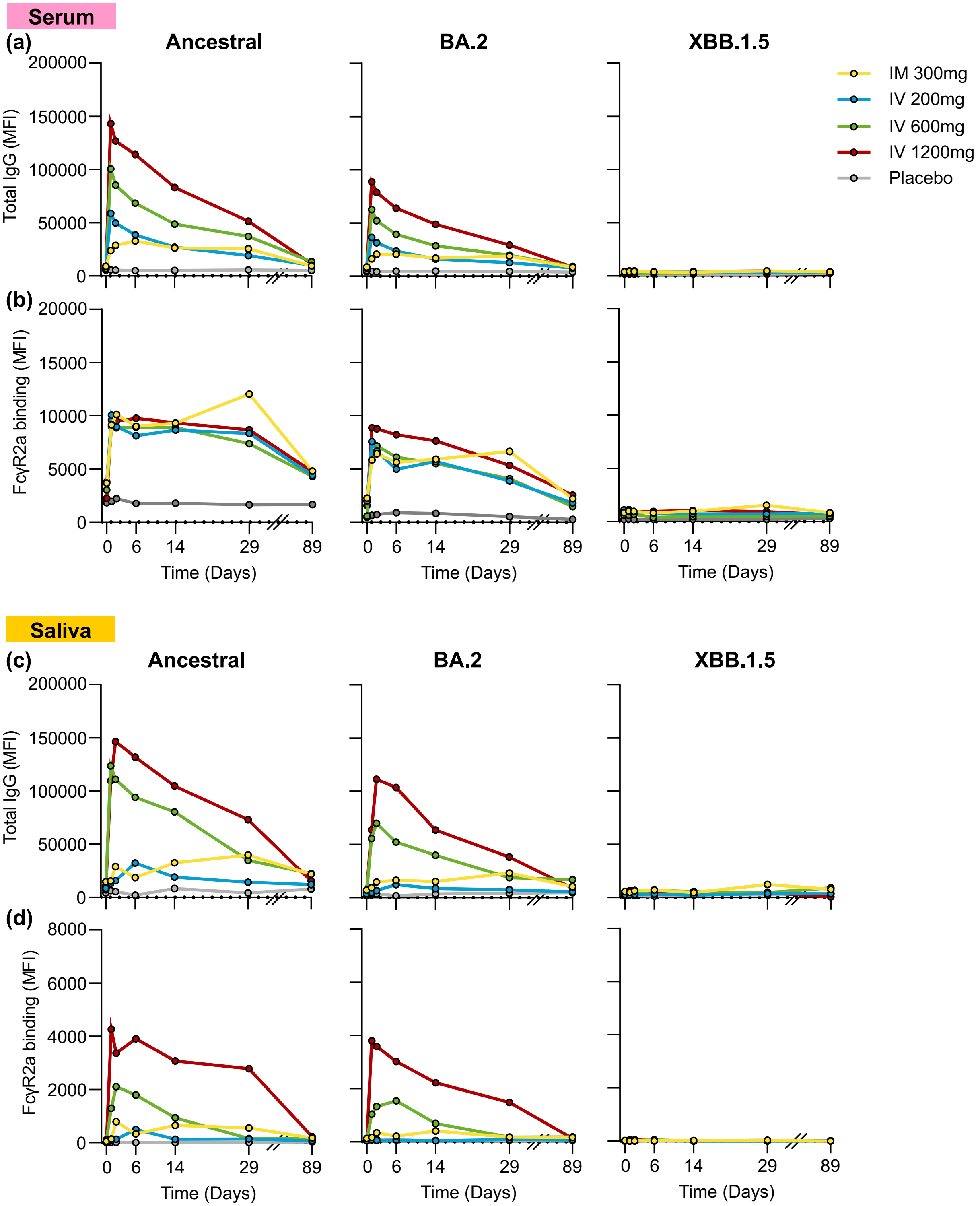

Supplementary Figure 6: Total IgG and FcγR2a-binding responses in serum and saliva. Ancestral, BA.2 and XBB.1.5 spike-specific **(a, c)** total IgG and **(b, d)** FcγR2a-binding antibody responses in **(a-b)** serum and **(c-d)** saliva across timepoints for each cohort measured via a bead-based multiplex assay. Dotted lines indicate the background responses of pre-pandemic samples. Median responses displayed for each cohort (n=6 for each IV and IM group, n=8 for placebo group). Assays were performed in duplicate.

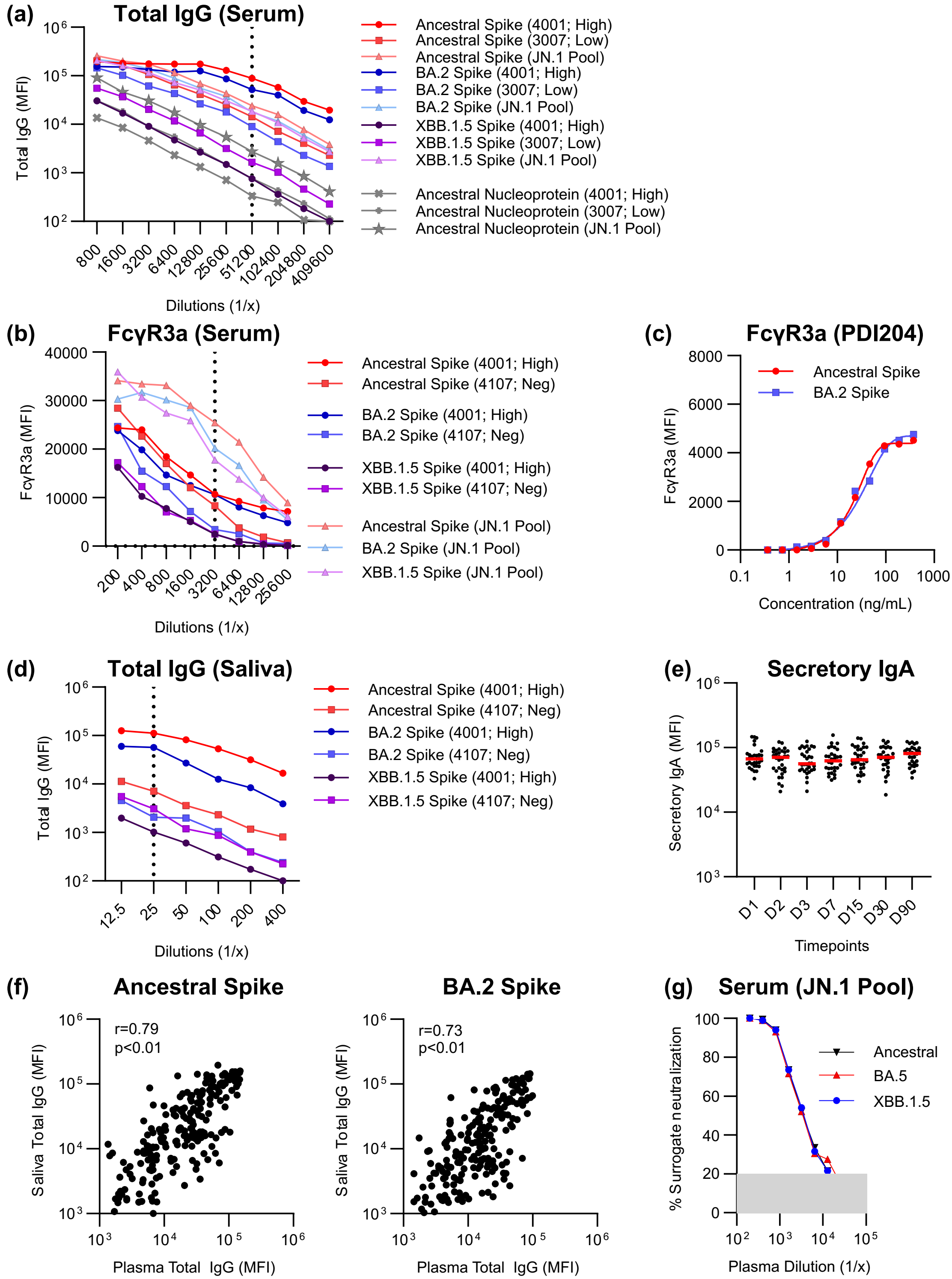

Supplementary Figure 7: Bead-based multiplex assay titration and validation. Titration of serum antibodies **(a)** total IgG and **(b)** FcγR3a binding to assay antigens using 3 different samples. **(c)** Titration of PDI204 spike-specific binding of FcγR3a. **(d)** Titration of salivary antibody total IgG binding to assay antigens using 2 different samples. **(e)** Levels of salivary secretory IgA found across all timepoints of the study. **(f)** Spearman correlations between spike-specific total IgG responses in saliva and serum. **(g)** Validation of surrogate viral neutralisation assay. Pooled plasma from JN.1 mRNA booster vaccinees was used as a positive control. Dotted line indicates the chosen assay dilution. Shaded region indicate the assay cutoff. Assays were performed in duplicate.
