# Supplementary material for: Randomised controlled phase I trial of a SARS‐CoV‐2 monoclonal antibody
Source: Clin Transl Immunology. 2026 Nov 4;15(11):e70126. doi: 10.1002/cti2.70126 (PMC13735506; doi:10.1002/cti2.70126)
Supplement: Supplementary file 3 [file CTI2-15-e70126-s002.docx]

**Supplementary Table 1: Participant demographics on study entry**

| **Characteristic** | **Total cohort (n=32)** | **IM 300mg (n=6)** | **IV 200mg (n=6)** | **IV 600mg (n=6)** | **IV 1200mg (n=6)** | **Placebo (n=8)** |
| --- | --- | --- | --- | --- | --- | --- |
| Age (years) | 33.0 (26.5, 37) | 32.0 (29.5, 40.8) | 26.5 (23.8, 65.0) | 35.0 (26.3, 61.3) | 33.5 (21.3, 41.3) | 35.0 (26.8, 50.5) |
| Sex (M/F), n (%) | 15 (45.5)/17 (54.5) | 4 (66.7)/2 (33.3) | 3 (50.0)/3 (50.0) | 2 (33.3)/4 (66.7) | 1 (16.7)/5 (83.30 | 5 (62.5)/3 (37.5) |
| Race, n (%) |  |  |  |  |  |  |
| White | 19 (59.4) | 2 (33.3) | 5 (83.3) | 4 (66.7) | 4 (66.7) | 4 (50) |
| Asian | 6 (18.8) | 2 (33.3) |  | 1 (16.7) | 1 (16.7) | 2 (25) |
| Other | 3 (9.4) | 1 (16.7) |  | 1 (16.7) | 1 (16.7) |  |
| American Indian / Alaska native* | 2 (6.3) | 1 (16.7) |  |  |  | 1 (12.5) |
| Black or African American | 1 (3.1) |  | 1 (16.7) |  |  |  |
| Native Hawaiian/ Other Pacific Islander | 1 (3.1) |  |  |  |  | 1 (12.5) |
| Weight (kg) | 75.5 (63.6, 86.7) | 74.5 (60.6, 88.7) | 73.5 (65.6, 87.1) | 81.2 (63.1, 94.4) | 66.4 (61.5, 85.3) | 76.5 (64.8, 90.9) |
| BMI | 25.6 (22.9, 28.9) | 25.3 (21.0, 28.2) | 26.0 (22.1, 28.6) | 29.8 (24.5, 31.5) | 24.7 (22.8, 27.6) | 24.1 (22.3, 28.2) |

All data presented as median (25^th^, 75^th^ percentiles) unless otherwise stated.

*includes persons having origins in any of the original peoples of North and South America (including Central America), and who maintains tribal affiliation or community attachment.

**Supplementary Table 2:** Post-treatment laboratory panels, physical examination, vital signs & ECG details

| **Category** | **Item** | **Adverse events** | **Comments** |
| --- | --- | --- | --- |
| Biochemistry | Albumin (Serum) | No adverse events |  |
|  | Alkaline Phosphatase | No adverse events |  |
|  | Alanine Aminotransferase (ALT) | **1 adverse event** | <2x ULN at day 4 post infusion in one participant receiving 200mg IV PDI204. Normal by day 14 |
|  | Aspartate Aminotransferase (AST) | **1 adverse event** |  |
|  | Bicarbonate (Serum) | No adverse events |  |
|  | Bilirubin Indirect | No adverse events |  |
|  | Bilirubin Total | No adverse events |  |
|  | Calcium (Serum) | No adverse events |  |
|  | Chloride (Serum) | No adverse events |  |
|  | Cholesterol | No adverse events |  |
|  | Conjugated Bilirubin | No adverse events |  |
|  | Creatinine | No adverse events |  |
|  | Creatine Phosphokinase (CPK) | No adverse events |  |
|  | Gamma-glutamyl transferase (GGT) | No adverse events |  |
|  | Glucose | No adverse events |  |
|  | HDL Cholesterol | No adverse events |  |
|  | Lipase (Serum) | **1 adverse event** | <5x ULN at day 4 post infusion in one participant receiving placebo in the 600mg IV group. Normal by day 6 |
|  | LDL Cholesterol | No adverse events |  |
|  | Estimated glomerular filtration rate (eGFR) | No adverse events |  |
|  | Phosphate | No adverse events |  |
|  | Potassium (Serum) | No adverse events |  |
|  | Sodium (Serum) | No adverse events |  |
|  | Total Protein | No adverse events |  |
|  | Triglyceride | No adverse events |  |
|  | Urea (Serum) | No adverse events |  |
| Coagulation | Activated Partial Thromboplastin Time (APTT) | No adverse events |  |
|  | Prothrombin Ratio INR | No adverse events |  |
|  | Prothrombin Time | No adverse events |  |
| Urinalysis | Bilirubin | No adverse events |  |
|  | Blood | No adverse events |  |
|  | Ketone Bodies | No adverse events |  |
|  | Micro Glucose | No adverse events |  |
|  | Micro Protein | No adverse events |  |
|  | Nitrite | No adverse events |  |
|  | pH | No adverse events |  |
|  | Specific Gravity | No adverse events |  |
|  | Urine appearance & colour | No adverse events |  |
|  | Urine Leukocyte Esterase | No adverse events |  |
|  | Urobilinogen | No adverse events |  |
| Haematology | Haemoglobin | No adverse events |  |
|  | Basophils | No adverse events |  |
|  | Basophils (Absolute) | No adverse events |  |
|  | Eosinophils | No adverse events |  |
|  | Eosinophils (Absolute) | No adverse events |  |
|  | Haemoglobin | No adverse events |  |
|  | Leucocytes | No adverse events |  |
|  | Lymphocytes | No adverse events |  |
|  | Lymphocytes (Absolute) | No adverse events |  |
|  | Mean cell haemoglobin (MCH) | No adverse events |  |
|  | Mean cell haemoglobin concentration (MCHC) | No adverse events |  |
|  | Mean cell volume (MCV) | No adverse events |  |
|  | Monocytes | No adverse events |  |
|  | Monocytes (Absolute) | No adverse events |  |
|  | Neutrophils | **1 adverse event** | One participant receiving 300mg IM PDI204 with baseline neutrophils of 2.4 x10^9^/L had neutrophil count 1.1-2.0x10^9^/L throughout follow-up |
|  | Neutrophils (Absolute) | No adverse events |  |
|  | Platelet Count | No adverse events |  |
|  | Packed cell volume (haematocrit) | No adverse events |  |
|  | Red Blood Cell Distribution Width (RDW) | No adverse events |  |
|  | Red Cell Count | No adverse events |  |
| Physical Examination | Head, Eyes, Ears, Nose and Throat | No adverse events |  |
|  | Lungs | No adverse events |  |
|  | Chest | No adverse events |  |
|  | Abdomen | No adverse events |  |
|  | Dermatological | **5 adverse events^** | 1 bruise at injection site day 2, resolved by day 4, in one participant receiving 600mg IV PDI204;  1 bruise from IV cannula at day 4, resolved by day 15 in participant receiving 1200mg IV PDI204;  1 redness at injection site and 1 contact dermatitis at injection site at day 1 in the same participant receiving 600mg PDI204, both resolved by day 6; 1 bandage-related contact dermatitis day 4, resolved by day 6, in one participant receiving 200mg IV PDI204 |
|  | Musculoskeletal | **1 adverse event** | Shoulder bursitis starting day 4, resolved by day 89 in one participant receiving 600mg IV PDI204 |
| Vital signs | Systolic bp | No adverse events |  |
|  | Diastolic bp | No adverse events |  |
|  | Heart rate | No adverse events |  |
|  | Respiratory | No adverse events |  |
|  | Body temperature (oral) | No adverse events |  |
| 12 lead standard ECG | Day 1 post-dose | No clinically significant findings |  |

Laboratory panels performed: days 1, 2, 4, 14, 29, 90

Physical examination performed: days 1, 2, 4, 6,14, 29, 59 & 89 post-dose

^ administration site conditions

Vital signs performed: 2/4/8/12/24 hours post-dose: days 2,4,6,14, 29, 59 &89 post-dose

**Supplementary table 3:** difference in serum neutralisation titres following PDI204 administration compared to baseline

|  | | | Median natural log difference (p-value) | | | | | |
| --- | --- | --- | --- | --- | --- | --- | --- | --- |
| Variant | **Dose** | **Route** | **Day 2** | **Day 3** | **Day 7** | **Day 15** | **Day 30** | **Day 90** |
| Ancestral | 0 | Placebo | -0.31 (0.38) | -0.17 (0.46) | -0.67 (0.3) | -0.34 (0.31) | -0.03 (0.95) | 0.36 (0.46) |
|  | 300 | IM | 2.11 (0.031) | 2.61 (0.031) | 2.35 (0.031) | 2.69 (0.031) | 1.85 (0.062) | 0.57 (0.063) |
|  | 200 | IV | 3.54 (0.031) | 3.77 (0.031) | 3.4 (0.031) | 3.06 (0.031) | 2.47 (0.031) | 0.69 (0.31) |
|  | 600 | IV | 4.77 (0.031) | 4.72 (0.031) | 3.74 (0.031) | 3.49 (0.031) | 2.39 (0.031) | 0.99 (0.16) |
|  | 1200 | IV | 7.58 (0.031) | 7.57 (0.031) | 6.53 (0.031) | 6.36 (0.031) | 6.28 (0.062) | 3.62 (0.031) |
| BA.5 | 0 | Placebo | -0.03 (0.95) | 0.1 (0.15) | -0.46 (0.11) | -0.27 (0.31) | -0.25 (0.46) | -0.24 (0.84) |
|  | 300 | IM | 0.21 (0.31) | 0.24 (0.31) | 0.32 (0.22) | 0.14 (0.56) | 0.04 (0.31) | 0.28 (0.69) |
|  | 200 | IV | 0.87 (0.063) | 0.7 (0.063) | 0.71 (0.031) | 0.46 (0.16) | 0.4 (0.44) | 0.15 (0.56) |
|  | 600 | IV | 1.17 (0.031) | 0.96 (0.031) | 0.72 (0.094) | 0.56 (0.22) | 0.47 (0.56) | 0.08 (1) |
|  | 1200 | IV | 0.92 (0.031) | 0.92 (0.063) | 0.82 (0.063) | 0.62 (0.094) | 0.01 (0.62) | -0.14 (1) |
| XBB.1.5 | 0 | Placebo | 0.05 (0.4) | 0.01 (0.4) | 0 (0.79) | 0.02 (1) | 0 (1) | 0.03 (0.67) |
|  | 300 | IM | 0.03 (0.59) | -0.03 (0.42) | -0.04 (0.42) | -0.07 (0.28) | -0.07 (1) | -0.04 (1) |
|  | 200 | IV | 0.01 (0.59) | -0.01 (0.59) | -0.01 (0.59) | -0.01 (0.42) | -0.03 (0.79) | 0.01 (0.79) |
|  | 600 | IV | 0.02 (0.44) | -0.01 (0.84) | 0.04 (0.56) | -0.1 (0.16) | -0.11 (0.063) | 0.13 (0.44) |
|  | 1200 | IV | -0.06 (0.56) | 0.06 (0.69) | -0.07 (0.31) | -0.07 (0.16) | -0.21 (0.062) | -0.3 (0.031) |

Statistical comparisons were done with the non-parametric Wilcoxon signed-rank test, comparing each timepoint to baseline (before PDI204 administration, Day 1).
